# Osteogenic Activity of New Dammarane‐Type Saponins From *Gynostemma pentaphyllum*

**DOI:** 10.1002/cbdv.71687

**Published:** 2026-09-10

**Authors:** Nguyen Tuan Duong, Tran Thi Thu Ha, Duong Thi Hai Yen, Do Thi Thao, Bui Huu Tai, Phan Van Kiem

**Affiliations:** ^1^ Bac Giang Agriculture and Forestry University, Viet Yen Bac Giang Vietnam; ^2^ Institute of Forestry and Sustainable Development University of Agriculture and Forestry Thai Nguyen University Thai Nguyen Vietnam; ^3^ Institute of Chemistry VAST Hanoi Vietnam; ^4^ Institute of Biology VAST Hanoi Vietnam

**Keywords:** cucurbitaceae, Gynopentanoside A, Gynopentanoside B, *Gynostemma pentaphyllum*, osteoclast activation

## Abstract

Five dammarane‐type saponins (**1**‐**5**) were isolated from the aerial parts of *Gynostemma pentaphyllum*, including two new compounds, 2α,3β,12β,19,20(*S*)‐pentahydroxydammar‐24‐ene 3‐*O*‐[β‐D‐glucopyranosyl‐(1→2)‐β‐D‐glucopyranoside]‐20‐*O*‐[β‐D‐xylopyranosyl‐(1→6)‐β‐D‐glucopyranoside] (**1**) and 12‐oxo‐2α,3β,20(*S*)‐trihydroxydammar‐24‐ene 3‐*O*‐(β‐D‐glucopyranoside)‐20‐*O*‐[β‐D‐xylopyranosyl‐(1→6)‐β‐D‐glucopyranoside] (**2**). The structures were elucidated through various spectroscopic analyses (IR, HRESIMS, 1D and 2D NMR). Compounds **1–3** and **5** exhibited osteogenic activity by stimulating alkaline phosphatase (ALP) activity, collagen synthesis, and calcium mineralization in mouse osteoblastic MC3T3‐E1 cells. Among these, compound **2** increased ALP activity by 1.30 ± 0.07‐ and 1.59 ± 0.16‐fold at 2 and 1 µg/mL, respectively, whereas compound **1** increased collagen synthesis and calcium mineralization by 1.14 ± 0.05‐ and 1.24 ± 0.06‐fold at 1 µg/mL, and compound **3** increased these parameters by 1.25 ± 0.07‐ and 1.14 ± 0.03‐fold at 2 µg/mL, respectively.

## Graphical abstract

Isolation and determination of two new and three known dammarane‐type glycosides from the aerial parts of *Gynostemma pentaphyllum* with their osteogenic activity via stimulation of alkaline phosphatase (ALP) activity, collagen synthesis, and calcium mineralization in mouse osteoblastic MC3T3‐E1 cells.

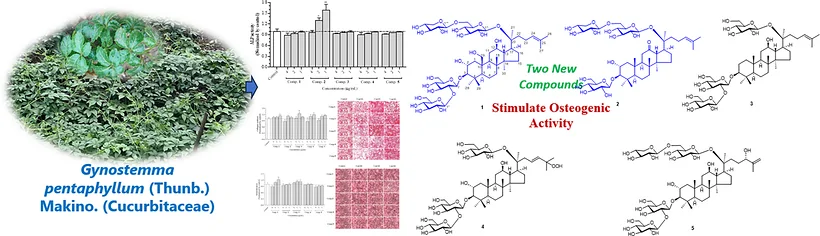

## 1 Introduction

Dammarane‐type saponins, which are most commonly found in *Panax* (Araliaceae) and *Gynostemma* (Cucurbitaceae) species, play important biological roles, including anti‐inflammatory, antioxidant, hepatoprotective, and immunomodulatory activities. Owing to these properties, they are widely used in the pharmaceutical, functional food, and cosmetic industries [1, 2, 3, 4, 5]. *Gynostemma pentaphyllum* (Thunb.) Makino, a herb used in traditional Chinese and Vietnamese medicine, is also known by several names, including Giảo Cổ Nam, Cổ Yếm, Thất Diệp Đảm, and Ngũ Diệp Sâm [3, 4, 5, 6]. This plant is known for various pharmacological effects, such as supporting diabetes treatment, reducing blood lipid levels, lowering blood pressure, exerting antioxidant effects, and alleviating stress and fatigue [7]. These pharmacological properties, particularly the antioxidant and anti‐inflammatory activities of *G. pentaphyllum*, may be relevant to bone metabolism and the maintenance of skeletal homeostasis. Promoting osteogenesis is of considerable biomedical interest because it plays an important role in bone regeneration, osteoporosis, and other conditions associated with impaired bone formation.

*Gynostemma pentaphyllum* and its major bioactive constituents, the dammarane‐type triterpenoid saponins known as gypenosides, have emerged as promising natural agents for bone regeneration and osteoporosis prevention [8, 9]. Although direct osteogenic studies remain relatively limited, most previous evidence has been obtained from crude extracts or gypenoside‐enriched preparations, whereas the osteogenic activities of individual dammarane‐type saponins remain largely unknown. Therefore, the isolation and evaluation of individual saponins are important for identifying bioactive constituents with osteogenic potential. Enhanced expression of osteogenic markers such as alkaline phosphatase (ALP), Runx2, osteocalcin (OCN), and osteopontin (OPN) has been reported following treatment with gypenoside‐rich preparations [9]. In addition to stimulating bone formation, gypenosides exhibit potent antioxidant and anti‐inflammatory activities that may protect osteoblasts from oxidative stress‐induced dysfunction and suppress osteoclast‐mediated bone resorption, thereby contributing to the maintenance of skeletal homeostasis. Recent pharmacological reviews have highlighted the broad regulatory effects of gypenosides on cellular signalling networks involved in oxidative stress, inflammation, and tissue regeneration, supporting their potential application as multifunctional agents for bone health [9, 10]. Based on the limited knowledge regarding the osteogenic activities of individual dammarane‐type saponins, this study aimed to isolate two new and three known dammarane‐type saponins from the aerial parts of *G. pentaphyllum* and evaluate their effects on ALP activity, collagen synthesis, and calcium mineralization in mouse osteoblastic MC3T3‐E1 cells. The objective was to identify individual saponins with osteogenic activity and provide a basis for further investigation of their biological effects.

## 2 Results and Discussion

### 2.1 Structure Elucidation

**FIGURE 1:**
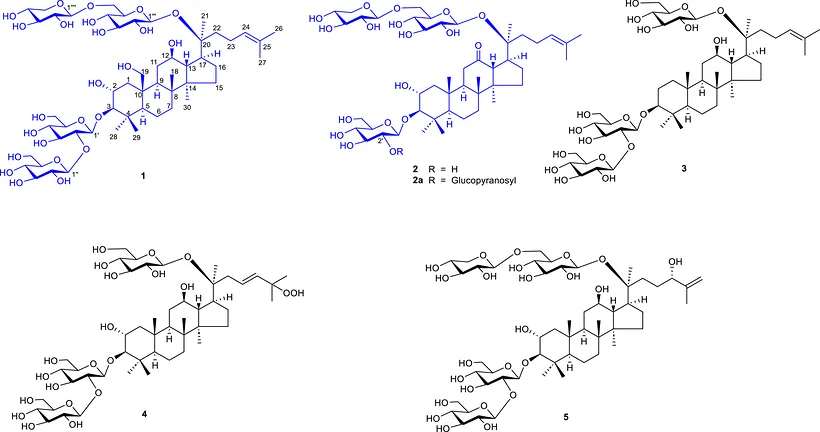
Chemical structures of compounds **1**–**5**.

**FIGURE 2:**
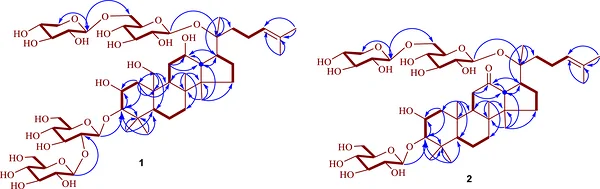
The key heteronuclear multiple bond correlation (HMBC) and ^1^H–^1^H correlated spectroscopy (COSY) correlations of compounds **1** and **2**.

**FIGURE 3:**
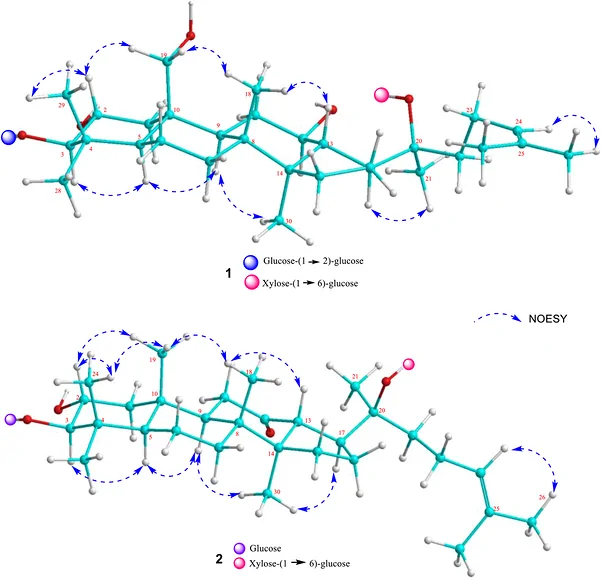
The key nuclear Overhauser effect spectroscopy (NOESY) correlations of compounds **1** and **2**.

Compound **1** (Figure 1) was isolated as a colorless amorphous powder, which exhibited absorption bands at 3400, 1450, and 1070 cm^−1^ on its infrared (IR) spectrum, indicating the presence of hydroxy, double bond, and C─*O*─C functional groups, respectively. High‐resolution electrospray ionization mass spectrometry (HRESIMS) showed a quasi‐molecular ion peak at *m/z* 1111.5907 [M+H]^+^ (calcd for C_53_H_91_O_24_^+^: 1111.5895, Δ = +1.1 ppm), determining the molecular formula to be C_53_H_90_O_24_ with nine degrees of unsaturation. The ^1^H‐nuclear magnetic resonance (NMR) spectrum of **1** suggested the presence of seven methyls [*δ*_H_ 1.08 (s, H_3_‐18), 1.38 (s, H_3_‐21), 1.71 (s, H_3_‐26), 1.65 (s, H_3_‐27), 1.16 (s, H_3_‐28), 0.98 (s, H_3_‐29), and 0.95 (s, H_3_‐30), each 3H)], one olefinic proton [*δ*_H_ 5.16 (t, 6.0 Hz, H‐24)], three methine carbinol protons [*δ*_H_ 4.01 (ddd, 12.0, 9.6, 4.8 Hz, H‐2), 3.03 (d, 9.6 Hz, H‐3), and 3.67 (m, H‐12)], one oxygenated methylene group [*δ*_H_ 3.90 (d, 12.0 Hz) and 3.82 (d, 12.0 Hz), H_2_‐19], and four anomeric protons [*δ*_H_ 4.47 (d, 7.8 Hz, H‐1ʹ), 4.78 (d, 7.8 Hz, H‐1ʹʹ), 4.59 (d, 7.8 Hz, H‐1ʹʹʹ), and 4.33 (d, 7.2 Hz, H‐1ʹʹʹʹ)]. The ^13^C‐NMR and the heteronuclear single quantum coherence (HSQC) spectra of **1** indicated 53 carbons, corresponding to 23 carbons from four sugar moieties and 30 carbons from a dammarane‐type triterpene skeleton, a framework previously reported from *G. pentaphyllum* [3, 4, 5, 6, 7]. In addition, seven methyls [*δ*_C_ 16.4 (C‐18), 22.4 (C‐21), 25.9 (C‐26), 18.0 (C‐27), 29.0 (C‐28), 17.2 (C‐29), and 17.7 (C‐20)], one double bond [*δ*_C_ 126.1 (C‐24) and 132.2 (C‐25)], three methine carbinol carbons [*δ*_C_ 68.7 (C‐2), 96.9 (C‐3), 71.8 (C‐12)], one oxygenated quaternary carbon [*δ*_C_ 85.0 (C‐20)], one oxygenated methylene carbon [*δ*_C_ 62.0 (C‐19)], and four anomeric carbons [*δ*_C_ 104.9 (C‐1′), 104.4 (C‐1′′), 98.1 (C‐1′′′), and 105.5 (C‐1′′′′) were observed. These data were similar to the corresponding data of 2α,3β,12β,20(S)‐tetrahydroxydammar‐24‐ene 3‐*O*‐[β‐D‐glucopyranosyl‐(1→2)‐β‐D‐glucopyranoside]‐20‐*O*‐[β‐D‐xylopyranosyl‐(1→6)‐β‐D‐glucopyrano‐side] (gypenoside LVI) [11], except 19‐methyl group was replaced by the oxygenated methylene group (Tables 1 and 2). This substitution also resulted in the C‐1 carbon shifting upfield, while the C‐10 carbon shifted downfield. These assignments were further confirmed by analysis of the ^1^H–^1^H COSY, HSQC, and HMBC spectra (Figure 2). HMBC correlations from H_2_‐19 (*δ*_H_ 3.90/3.82) to C‐1 (*δ*_C_ 43.0)/C‐5 (*δ*_C_ 57.4)/C‐9 (*δ*_C_ 52.2)/C‐10 (*δ*_C_ 43.9) indicated the presence of a 19‐hydroxyl group. Protons H_3_‐28 (*δ*_H_ 1.16) and H_3_‐29 (*δ*_H_ 0.98) showed correlations to C‐3 (δ_C_ 96.9), C‐4 (δ_C_ 41.6), and C‐5 (δ_C_ 57.4), while proton H_2_‐1 correlated to C‐2 (δ_C_ 68.7) and C‐3 (δ_C_ 96.9), confirming that C‐2 and C‐3 were oxygenated. The ^1^H–^1^H COSY cross peaks of H‐9/H‐11/H‐12/H‐13/H‐17/H‐22/H‐23 together with HMBC correlations from H_3_‐30 (*δ*_H_ 0.95) to C‐8 (*δ*_C_ 41.0)/C‐13 (*δ*_C_ 49.9)/C‐14 (*δ*_C_ 52.5), from H_3_‐21 (*δ*_H_ 1.38) to C‐17 (*δ*_C_ 53.0)/C‐20 (*δ*_C_ 85.0)/C‐22 (*δ*_C_ 36.8), and from H_3_‐26 (*δ*_H_ 1.71)/H_3_‐27 (*δ*_H_ 1.65) to C‐24 (*δ*_C_ 126.1)/C‐25 (*δ*_C_ 132.2) indicated that C‐12 and C‐20 were oxygenated, and a double bond was located between C‐24 and C‐25. The sugar moieties were the same as those of gypenoside LVI [9] as evident by HSQC and ^1^H–^1^H COSY spectra, and by HMBC correlations from H‐1′′ (*δ*_H_ 4.78) to C‐1′ (*δ*_C_ 80.6), from H‐1′ (*δ*_H_ 4.47) to C‐3 (*δ*_C_ 96.9), from H‐1′′′′ (*δ*_H_ 4.33) to C‐6′′′ (*δ*_C_ 70.1), and from H‐1′′′ (*δ*_H_ 4.59) to C‐20 (*δ*_C_ 85.0), as well as by ^1^H–^1^H COSY analyzing of each sugar moiety (Figure 2). The large ^3^ *J*_1′,2′_ values (7.8 Hz) of all the anomeric protons suggested *β*‐glycosidic linkages. Proton H‐3 appeared at *δ*_H_ 3.03 as a doublet with a large ^3^ *J*_2,3_ coupling constant (9.6 Hz), suggesting that both H‐2 and H‐3 have *axial* orientations. This was further confirmed by NOE cross‐peaks between H‐3 (*δ*_H_ 3.03) and H‐5 (*δ*_H_ 0.96), as well as between H_2_‐19 (*δ*_H_ 3.90/3.82) and H‐2 (*δ*_H_ 4.01). Proton H‐12 (*δ*_H_ 3.67) showed NOE cross‐peaks with H_3_‐30 (*δ*_H_ 0.95) and H‐17 (*δ*_H_ 2.30), indicating an *α/axial* orientation for H‐12 (Figure 3). The stereochemistry of dammarane skeleton was supported by NOESY correlations of H_3_‐29 (*δ*_H_ 0.98)/ H_2_‐19 (*δ*_H_ 3.90 and 3.82)/ H_3_‐18 (*δ*_H_ 1.08)/ H‐13 (*δ*_H_ 1.77) and H_3_‐28 (*δ*_H_ 1.16)/ H‐5 (*δ*_H_ 0.96)/ H‐9 (*δ*_H_ 1.55)/ H_3_‐30 (*δ*_H_ 0.95)/ H‐17 (*δ*_H_ 2.30). The identical carbon chemical shift values at C‐20 and the anomeric carbon Glc C‐1‴ between compound **1** (*δ*_C‐20_ = 85.0; *δ*_C‐1‴_ = 98.1) and ginsenoside Rd (**3**: *δ*_C‐20_ = 84.9; *δ*_C‐1‴_ = 98.3) in the same NMR solvent measurement (CD_3_OD) indicated that both compounds shared the 20*S* absolute configuration. Additionally, the 20*S*‐configuration was further demonstrated by the carbon chemical shifts of C‐17 and C‐22, as previously described [12]. The carbon chemical shifts of C‐17 (*δ*_C_ 53.0) and C‐22 (*δ*_C_ 36.8) in compound **1** closely resembled those of 20*S*‐ginsenoside Rg3 (*δ*_C_ 54.9 and 36.0, respectively) and differed significantly from those of 20*R*‐ginsenoside Rg3 (*δ*_C_ 50.7 and 43.3, respectively) [12].

**TABLE 1:** Nuclear magnetic resonance (NMR) spectral data for the aglycones of compounds **1** and **2** in CD_3_OD.

No.	1		2	
	*δ*_C_ ^a^	*δ*_H_ (*J* in Hz)^b^	*δ*_C_ ^a^	*δ*_H_ (*J* in Hz)^b^
1	43.0	0.82 (t, 12.0) 2.65 (dd, 12.0, 4.8)	47.5	0.92 (t, 10.8) 1.97 (dd, 10.8, 3.6)
2	68.7	4.01 (ddd, 12.0, 9.6, 4.8)	68.1	3.75 (ddd, 10.8, 9.6, 3.6)
3	96.0	3.03 (d, 9.6)	95.7	3.03 (d, 9.6)
4	41.6	—	41.9	—
5	57.4	0.96 (br d, 11.4)	57.0	0.93 (d, 11.4)
6	18.9	1.50 (m)/1.60 (m)	19.4	1.60 (m)/1.68 (m)
7	35.9	1.34 (m)/1.60 (m)	35.4	1.44 (m)/1.58 (m)
8	41.0	—	41.6	—
9	49.9	1.55 (m)	56.1	1.79 (dd, 12.6, 3.6)
10	43.9	—	39.4	—
11	32.8	1.66 (m)/2.00 (m)	40.9	2.49 (t, 12.6)/2.17 (dd, 12.6, 3.6)
12	71.8	3.67 (m)	214.9	—
13	49.9	1.77 (m)	57.2	3.32^c^
14	52.5	—	57.4	—
15	31.7	1.08 (m)/1.62 (m)	33.0	1.16 (m)/1.82 (m)
16	27.3	1.36 (m)/1.92 (m)	25.0	1.76 (m)/1.93 (m)
17	53.0	2.30 (m)	43.1	2.52 (m)
18	16.4	1.08 (s)	16.3	1.29 (s)
19	62.0	3.90 (d, 12.0) 3.82 (d, 12.0)	17.8	1.07 (s)
20	85.0	—	82.7	—
21	22.4	1.38 (s)	22.8	1.14 (s)
22	36.8	1.55 (m)/1.82 (m)	40.6	1.64 (m)
23	23.9	2.05 (m)/2.16 (m)	24.6	2.02 (q, 7.2)
24	126.1	5.16 (t, 6.0)	126.0	5.13 (t, 7.2)
25	132.2	—	132.0	—
26	25.9	1.71 (s)	25.9	1.71 (s)
27	18.0	1.65 (s)	17.9	1.65 (s)
28	29.0	1.16 (s)	28.6	1.15 (s)
29	17.2	0.98 (s)	17.8	0.94 (s)
30	17.7	0.95 (s)	17.2	0.78 (s)

^a^ 150 MHz.

^b^ 600 MHz.

^c^ Overlapped signals.

**TABLE 2:** Nuclear magnetic resonance (NMR) spectral data for the sugar moieties of compounds **1** and **2** in CD_3_OD.

No.	1		2	
	*δ*_C_ ^a^	*δ*_H_ (*J* in Hz)^b^	*δ*_C_ ^a^	*δ*_H_ (*J* in Hz)^b^
3‐*O*‐β‐D‐glucose			3‐*O*‐β‐D‐glucose	
1ʹ	104.9	4.47 (d, 7.8)	106.5	4.36 (d, 7.8)
2ʹ	80.6	3.71 (dd, 9.0, 7.8)	75.5	3.28 (dd, 9.0, 7.8)
3ʹ	78.7	3.61 (t, 9.0)	78.2	3.35 (t, 9.0)
4ʹ	72.1	3.22^c^	71.5	3.37 (t, 9.0)
5ʹ	78.0	3.21^c^	78.1	3.35 (m)
6ʹ	62.4	3.68 (dd, 12.0, 5.4) 3.88^c^	62.4	3.68 (dd, 12.0, 5.4) 3.88 (dd, 12.0, 1.8)
2ʹ‐*O*‐β‐D‐glucose				
1ʹʹ	104.4	4.77 (d, 7.8)		
2ʹʹ	76.2	3.25^c^		
3ʹʹ	78.6	3.35^c^		
4ʹʹ	71.5	3.35^c^		
5ʹʹ	78.0	3.28 (ddd, 9.0, 5.4, 2.4)		
6ʹʹ	63.2	3.63 (dd, 12.0, 5.4) 3.85 (dd, 12.0, 2.4)		
20‐*O*‐β‐D‐glucose			20‐*O*‐β‐D‐glucose	
1ʹʹʹ	98.1	4.59 (d, 7.8)	98.4	4.46 (d, 7.8)
2ʹʹʹ	75.3	3.15 (dd, 9.0, 7.8)	75.6	3.12 (dd, 9.0, 7.8)
3ʹʹʹ	78.3	3.35^c^	78.6	3.35 (t, 9.0)
4ʹʹʹ	71.2	3.37^c^	71.4	3.37 (t, 9.0)
5ʹʹʹ	76.2	3.45 (m)	76.3	3.37 (m)
6ʹʹʹ	70.1	3.75 (dd, 11.4, 4.8) 4.03 (dd, 11.4, 1.8)	70.1	3.77 (dd, 11.4, 4.8) 4.01 (dd, 11.4, 1.8)
6ʹʹʹ‐*O*‐β‐D‐xylose			6ʹʹʹ‐*O*‐β‐D‐xylose	
1ʹʹʹʹ	105.5	4.33 (d, 7.8)	105.5	4.31 (d, 7.2)
2ʹʹʹʹ	74.8	3.22 (dd, 9.0, 7.8)	74.8	3.22 (dd, 9.0, 7.8)
3ʹʹʹʹ	77.5	3.34^c^	77.5	3.33 (t, 9.0)
4ʹʹʹʹ	71.2	3.50 (m)	71.2	3.50 (m)
5ʹʹʹʹ	66.8	3.20 (dd, 11.4, 9.6) 3.88 (dd, 11.4, 4.8)	66.8	3.20 (dd, 11.4, 9.6) 3.88 (dd, 11.4, 4.8)

^a^ 150 MHz.

^b^ 600 MHz.

^c^ Overlapped signals.

Acid hydrolysis of **1** yielded D‐glucose and D‐xylose, which were identified via thin‐layer chromatography (TLC) and optical rotation in comparison with authentic samples [13, 14]. Thus, compound **1** was determined to be 2α,3β,12β,19,20(*S*)‐pentahydroxydammar‐24‐ene 3‐*O*‐[β‐D‐glucopyranosyl‐(1→2)‐β‐D‐glucopyranoside]‐20‐*O*‐[β‐D‐xylopyranosyl‐(1→6)‐β‐D‐glucopyranoside], a new compound named gynopentanoside A. Compound **2** was obtained as a colorless amorphous powder. Its IR spectrum suggested the presence of hydroxy (3400 cm^−1^), double bond (1500 cm^−1^), and C‐*O*‐C (1070 cm^−1^) functional groups. Compound **2** had the molecular formula C_47_H_78_O_18_ (nine degrees of unsaturation) as determined by the HRESIMS (found *m/z* 931.5261 [M + H]^+^, calcd for C_47_H_79_O_18_^+^: 931.5261, Δ = 0 ppm). The ^1^H, ^13^C NMR, and HSQC spectra of **2** were characteristic of a dammarane‐type triterpene saponins, showing signals for eight tertiary methyl groups [*δ*_C_/*δ*_H_: 16.3 (C‐18)/1.29 (s), 17.8 (C‐19)/1.07 (s), 22.8 (C‐21)/1.14 (s), 25.9 (C‐26)/1.71 (s), 17.9 (C‐27)/1.65 (s), 28.6 (C‐28)/1.15 (s), 17.8 (C‐29)/0.94 (s), and 17.2 (C‐30)/0.78 (s)], one Δ^24^ double bond [*δ*_C_/*δ*_H_: 126.0 (C‐24)/5.13 (t, 7.2) and 132.0 (C‐25)], two methine carbinol groups [*δ*_C_/*δ*_H_: 68.1 (C‐2)/3.75 (ddd, *J* = 10.8, 9.6, 3.6 Hz) and 95.7 (C‐3)/3.03 (d, *J* = 9.6 Hz)], one ketone group [*δ*_C_ 214.9 (C‐12)], and three anomeric groups [*δ*_C_/*δ*_H_: 106.5 (C‐1′)/4.36 (d, *J* = 7.8 Hz), 98.4 (C‐1′′′)/4.46 (d, *J* = 7.8 Hz), and 105.5 (C‐1′′′′)/4.31 (d, *J* = 7.2 Hz)] (Tables 1 and 2) [3, 4, 5, 6, 7, 13]. Similar to compound **1**, the signal assigned to C‐6′′′ of the 20‐*O*‐Glc unit moiety shifted downfield by approximately 7.4 ppm, appearing at δ_C_ 70.1, suggesting that a xylose unit (*δ*_C_: 105.5, 74.8, 77.5, 71.2, 66.8) was linked to C‐ C‐6′′′ via an ether linkage. This was further confirmed by HMBC correlation from H‐1′′′′ (*δ*_H_ 4.31) to C‐6′′′ (*δ*_C_ 70.1). The xylose‐(1→6)‐glucose moiety was shown to be attached to C‐20 as evidenced by HMBC correlation from H‐1′′′ (*δ*_H_ 4.46) to C‐20 (*δ*_C_ 82.7). Analysis NMR data of **2**, in comparison with the corresponding data for 12‐oxo‐2α,3β,20(S)‐trihydroxydammar‐24‐ene 3‐*O*‐[β‐D‐glucopyranosyl‐(1→2)‐β‐D‐glucopyranoside]‐20‐*O*‐[β‐D‐xylopyranosyl‐(1→6)‐β‐D‐glucopyranoside] (**2a**), a compound previously isolated from the same source, *G. Pentaphyllum* [15], revealed that the sugar moieties attached to C‐3 has lost one glucose unit (Table 2). This was further supported by the C‐2′ signal shifting upfield by 5.1 ppm, appearing at *δ*_C_ 75.5, and the observation of an HMBC correlation from H‐1′ (*δ*_H_ 4.36) to C‐3 (*δ*_C_ 95.7). The large ^3^ *J*_2,3_ coupling constant (9.6 Hz) suggested that both Hβ‐2 and Hα‐3 have *axial* orientations. The large ^3^ *J*_1′,2′_ values (7.2–7.8 Hz) of the anomeric protons indicated the *β*‐form of the glycosidic linkages. Acid hydrolysis of **2** yielded D‐glucose and D‐xylose, which were identified by TLC and optical rotation in comparison with authentic samples [13, 14]. Thus, compound **2** was determined to be 12‐oxo‐2α,3β,20(S)‐trihydroxydammar‐24‐ene 3‐*O*‐(β‐D‐glucopyranoside)‐20‐*O*‐[β‐D‐xylopyranosyl‐(1→6)‐β‐D‐glucopyranoside], a new compound named as gynopentanoside B.

Three known compounds were identified as ginsenoside Rd (**3**) [16], gypenoside GD5 (**4**) [17], 2α,3β,12β,20(*S*),24(*S*)‐pentahydroxy‐dammar‐25‐ene 3‐*O*‐β‐D‐glucopyranosyl‐(1→2)‐β‐D‐glucopyranosyl‐20‐*O*‐β‐D‐xylopyranosyl‐(1→6)]‐β‐D‐glucopyranoside (**5**) (Figures S20–S30 and Tables S1–S3) [18].

### 2.2 Evaluation of Osteogenic Activity

**FIGURE 4:**
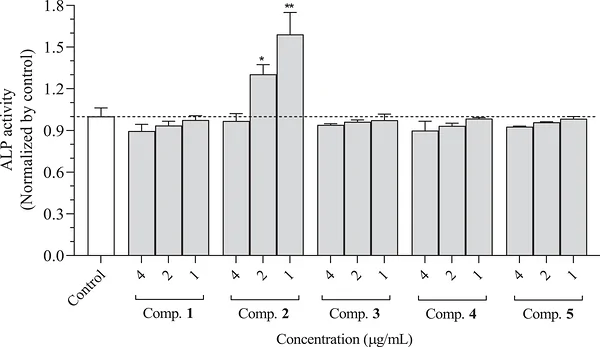
ALP activity of compounds **1**–**5** in osteoblastic MC3T3‐E1 cells (*n* = 3, ^**^ *p* < 0.01, ^*^ *p* < 0.05).

**FIGURE 5:**
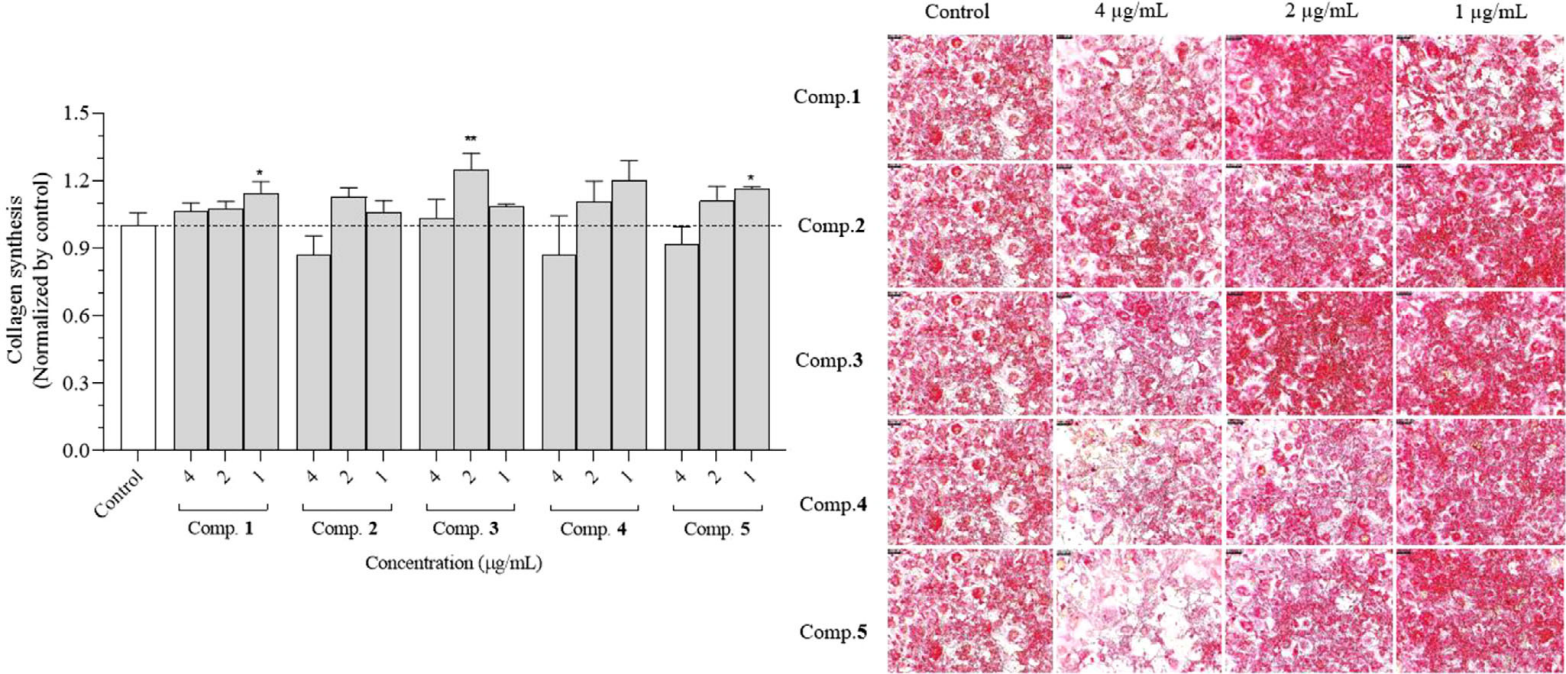
Collagen quantification and collagen staining in osteoblastic MC3T3‐E1 cells treated with compounds **1**–**5** (*n* = 3, ^**^ *p* < 0.01, ^*^ *p* < 0.05). An image of the control is provided for each compound for comparison.

**FIGURE 6:**
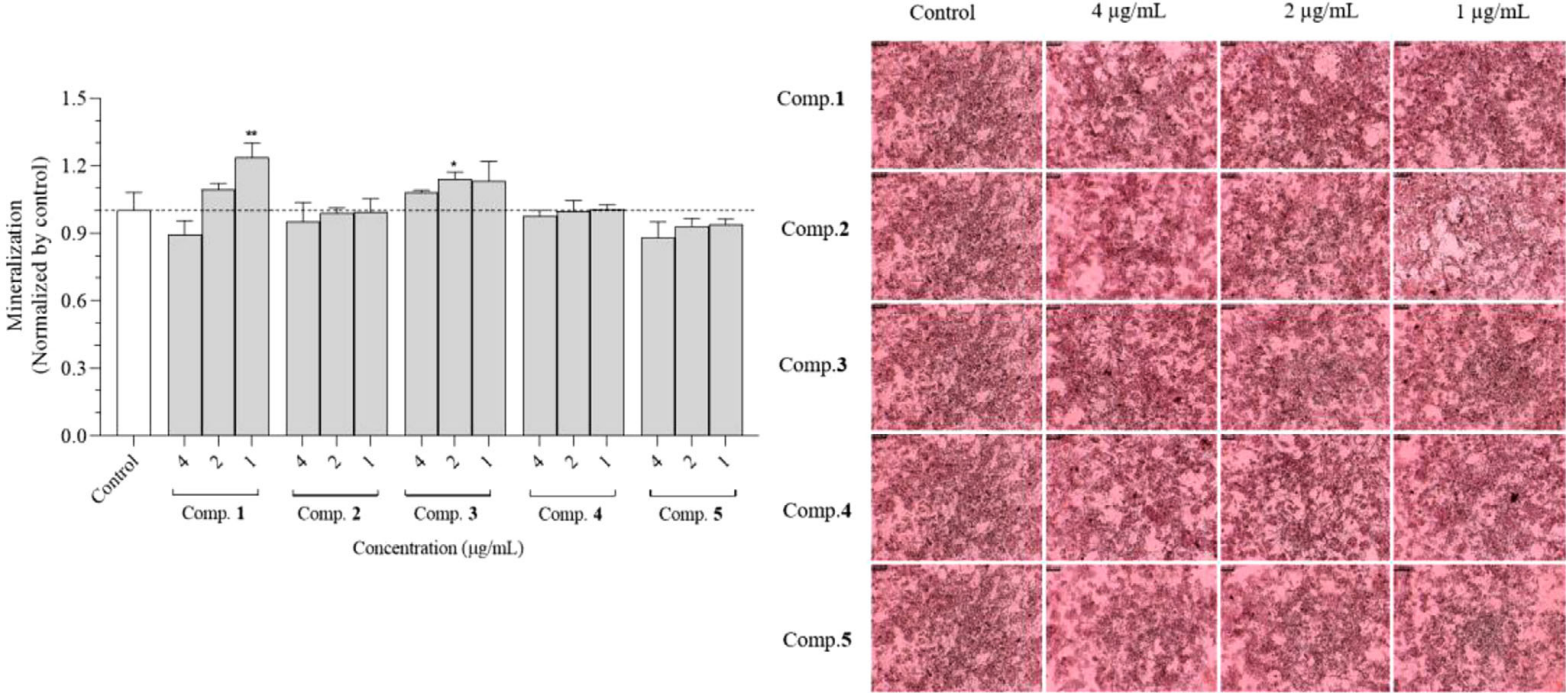
Quantification and Alizarin red staining for calcium mineralization in osteoblastic MC3T3‐E1 cells treated with compounds **1**–**5** (*n* = 3, ^**^ *p* < 0.01, ^*^ *p* < 0.05). An image of the control is repeatedly provided for each compound for comparison.

Osteogenic activity refers to the process by which osteoblasts and their precursor cells differentiate, produce bone extracellular matrix, and promote mineral deposition to form new bone tissue. This process is essential for skeletal development, bone remodeling, and fracture repair. It plays a critical role in maintaining bone strength and structural integrity. During osteogenesis, increased collagen production reflects active matrix formation, which provides the structural framework necessary for subsequent mineralization. On the other hand, osteoblasts increase ALP expression and activity to promote bone mineralization. ALP also helps reduce the concentration of mineralization inhibitors such as pyrophosphate. At last stages, osteoblasts deposit calcium and phosphate ions into the collagen‐rich extracellular matrix that provides bone with its strength and rigidity. Therefore, osteogenic activity is commonly evaluated through markers of osteoblast differentiation and function, including ALP activity, collagen synthesis, and calcium mineralization, whereas enhanced osteogenesis is a key therapeutic strategy for promoting bone regeneration and preventing bone loss [19]. The osteogenic activity of compounds **1–5** was evaluated based on their stimulatory effects on ALP activity, collagen synthesis, and calcium mineralization as previously described [20, 21, 22]. First, the proliferation of mouse osteoblastic MC3T3‐E1 cells in the presence of the compounds (100, 20, 4, and 0.8 µg/mL) was examined. No cytotoxic effects were observed at concentrations of 4 and 0.8 µg/mL after 3 days of treatment; however, significant cytotoxic effects were observed at higher concentrations of 20 and 100 µg/mL (Figure S31). Therefore, the effects of compounds **1–5** on ALP activity, collagen synthesis, and calcium mineralization were evaluated at lower concentrations of 4, 2, and 1 µg/mL. Compound **2** significantly stimulated ALP activity at concentrations of 2 and 1 µg/mL, increasing activity by 1.30 ± 0.07 (*p* < 0.05) and 1.59 ± 0.16‐fold (*p* < 0.01), respectively, compared with the control (Figure 4). Compounds **1** and **3**–**5** did not clearly promote ALP activity at any of the tested concentrations. As shown in Figure 5, compounds **1**, **3**, and **5** significantly increased collagen synthesis. In particular, at a concentration of 1 µg/ml, compounds **1** and **5** increased collagen synthesis by 1.14 ± 0.05‐and 1.17 ± 0.08‐fold compared with the control (*p *< 0.05). Meanwhile, compound **3** increased collagen synthesis by 1.25 ± 0.07‐fold at a concentration of 2 µg/mL (*p* < 0.01). With respect to calcium mineralization, our results indicated that compound **1** (1 µg/mL) and compound **3** (2 µg/mL) significantly enhanced mineralization by 1.24 ± 0.06‐ and 1.14 ± 0.03‐fold, respectively, compared with the control. In summary, compounds **1–5** modulated osteogenic markers in MC3T3‐E1 cells under the evaluated conditions, with compound **2** stimulating ALP activity at 1 and 2 µg/mL, compounds **1** (1 µg/mL) and **3** (2 µg/mL) enhancing both collagen synthesis and calcium mineralization, and compound **5** (1 µg/mL) enhancing collagen synthesis (Figure 6). The distinct responses may be explained by the different stages of osteoblastic differentiation targeted by the compounds. ALP is an early marker of osteogenic differentiation and is involved in the initiation of matrix mineralization, whereas collagen synthesis and calcium deposition are hallmarks of the later stages of osteoblast maturation [23]. Thus, the increased ALP activity induced by compound **2** may indicate the promotion of early osteoblastic differentiation but may not be sufficient to drive matrix production or mineralization under the present experimental conditions. In contrast, compounds **1** and **3** may preferentially promote later stages of osteogenic differentiation, leading to increased collagen synthesis and calcium deposition despite no significant increase in ALP activity, as ALP expression and activity tend to decline as mineralized tissue matures.

The closely related dammarane‐type saponin analogues, ginsenosides, have recently been shown to promote osteoblast differentiation and function. Several ginsenosides, including Rb1, Rg1, and Rd, enhance osteogenic activity by stimulating signaling pathways involved in bone formation, particularly the BMP‐2 and Wnt signaling pathways [24]. The osteogenic activities observed for compounds **1–5** further support the beneficial effects of these dammarane saponins, specifically the gypenosides derived from *G. pentaphyllum*. Osteoporosis is a common skeletal disorder characterized by reduced bone mass and deterioration of bone microarchitecture, resulting in increased bone fragility and a higher risk of fractures. This disease develops when the balance between bone formation by osteoblasts and bone resorption by osteoclasts is disrupted, leading to progressive bone loss. Current osteoporosis treatments, therefore, aim to restore bone homeostasis by either inhibiting bone resorption or stimulating bone formation [25]. Despite the promising in vitro osteogenic activity, the present study has several limitations. Osteogenic activity was evaluated in only one osteoblastic cell line and without a positive osteogenic control. In addition, osteogenic markers, including Runx2, Sp7/Osx, Col1a1, Alpl, Bglap, and Spp1, together with the BMP‐2 and Wnt/β‐catenin signaling pathways, were not investigated. The effects of these compounds on osteoclast‐mediated bone resorption and in vivo bone formation also remain unknown. The isolated compounds exhibited osteogenic activity in vitro and may represent promising lead compounds for further investigation. Therefore, additional mechanistic studies and in vivo validation are required before their potential application in the prevention or treatment of osteoporosis can be established.

## 3 Conclusions

Phytochemical investigation of the aerial parts of *G. pentaphyllum* led to the isolation of five dammarane‐type saponins, including two previously unreported compounds (**1** and **2**). Biological evaluation revealed that compounds **1–3** and **5** exhibited preliminary pro‐osteogenic activity in MC3T3‐E1 osteoblastic cells as reflected by changes in ALP activity, collagen synthesis, and calcium mineralization under the experimental conditions evaluated. Compound **2** increased ALP activity, whereas compounds **1** and **3** enhanced collagen synthesis and calcium mineralization, while compound **5** selectively increased collagen synthesis. These findings provide preliminary in vitro evidence that selected dammarane‐type saponins from *G. pentaphyllum* can modulate osteogenic markers in MC3T3‐E1 cells and warrant further investigation.

## 4 Experimental Section

### 4.1 General

The equipment used for this study is shown in the Supporting Information.

### 4.2 Plant Material

The aerial parts of *G. pentaphyllum* were collected in Tung Vai, Tuyen Quang, Vietnam, in March 2025 and identified by Dr. Do Hoang Chung, Thai Nguyen University of Agriculture and Forestry. A voucher specimen (NCCT‐P200) was deposited at Thai Nguyen University of Agriculture and Forestry.

### 4.3 Extraction and Isolation

The detailed extraction and isolation of compounds **1**–**5** were shown in the Supporting Information.

#### 4.3.1 Gynopentanoside A (**1**)

A colorless amorphous powder; ${\left[\alpha \right]}_{D}^{25}$: +18.0 (*c* 0.1, MeOH); HRESIMS *m/z* 1111.5907 [M + H]^+^ (calcd for C_53_H_91_O_24_^+^: 1111.5895, Δ = +1.1 ppm). ^1^H NMR (CD_3_OD, 600 MHz) and ^13^C NMR (CD_3_OD, 150 MHz) data are shown in Tables 1 and 2 (Figures S1–S9).

#### 4.3.2 Gynopentanoside B (**2**)

A colorless amorphous powder; ${\left[\alpha \right]}_{D}^{25}$: +14.5 (*c* 0.1, MeOH); HRESIMS *m/z* 931.5261 [M + H]^+^ (calcd for C_47_H_79_O_18_^+^: 931.5261, Δ = 0 ppm). ^1^H NMR (CD_3_OD, 600 MHz) and ^13^C NMR (CD_3_OD, 150 MHz) data are shown in Tables 1 and 2 (Figures S10–S18).

The NMR data of compounds **3**–**5** were shown in Supporting Information (Tables S1–S3 and Figures S19–S30).

#### 4.3.3 Acid Hydrolysis of Compounds **1** and **2**

Acid hydrolysis of compounds **1** and **2** was the same as described in previous work [13, 14], referring to Supporting Information.

### 4.4 Cell Culture

Mouse osteoblastic MC3T3‐E1 cells (ATCC, RRID: CRL‐2593) were seeded (density of 1×10^4^ cells/ml) and cultured in growth culture medium containing alpha‐MEM supplemented with 10% fetal bovine serum and 1% penicillin‐streptomycin at 37°C in a humidified atmosphere (5% CO_2_). At 80% confluence, the cells were cultured in growth culture medium supplemented with 10 mM β‐glycerophosphate and 50 µg/ml ascorbic acid (osteogenic differentiation medium).

### 4.5 Cell Proliferation

Cell proliferation was determined by MTT assay. Briefly, cells (density of 1×10^4^ cells/well in a 96‐well plate) were incubated with/without compounds (concentrations of 4 and 0.8 µg/mL) in growth medium for 72 h at 37°C in a humidified atmosphere (5% CO_2_). After that, 20 µl of MTT solution (5 mg/mL) was added to the cell cultures and incubated at 37°C for 4 h. Formazan crystals were dissolved in DMSO, and absorbance was measured at 490 nm using a BioTek ELx800 ELISA Plate Reader.

### 4.6 ALP Activity

The cells were incubated with/without compounds (concentrations of 1, 2, and 4 µg/ml) in osteogenic differentiation media. The medium containing compounds was changed every 2 days. ALP activity was measured after 7 days using the ALP Assay Kit (Abcam, Cambridge, England), according to the manufacturer's instructions.

#### 4.6.1 Collagen Measurement

The cells were incubated with/without compounds (concentrations of 1, 2, and 4 µg/ml) in osteogenic differentiation media for 10 days. The plate was washed with PBS and fixed with Bouin's fluid for 1 h. After that, the wells were washed with water and allowed to dry at 37°C in a humidified atmosphere for 24 h. After drying, the wells were stained with 0.1% Sirius red dye in picric acid for 1 h and then washed with HCl solution (0.01 M) to remove the unbound dye. Finally, the bound collagen was dissolved in 0.1 M NaOH solution, and absorbance was read at 550 nm using a microplate reader.

### 4.7 Extracellular Matrix Calcium Deposit Staining Assay

The cells were incubated with/without compounds (concentrations of 1, 2, and 4 µg/ml) in osteogenic differentiation media for 15 days. After that, the cells were washed with PBS twice and then fixed with ethanol for 1 h. The cells were stained with Alizarin red S solution (40 mM, pH = 4.4) for 15 min and then rinsed with deionized water twice. The bound calcium was dissolved in 10% cetylpyridinium chloride solution for 15 min, and absorbance was read at 561 nm using a microplate reader.

### 4.8 Statistical Analyses

Values for cell proliferation, ALP activity, collagen synthesis, and calcium mineralization are presented as mean±SD (*n* = 3). Data analysis was performed by Microsoft Excel using Student's *t*‐test, F’‐test, one‐way analysis of variance (ANOVA), and Bonferroni's Multiple Comparison Test. Significance was detected between the treated samples versus control at the level of *p* < 0.05.

## Author Contributions

**Phan Van Kiem**: writing – original draft, writing – review and editing, data curation, formal analysis, supervision. **Tran Thi Thu Ha**: conceptualization, investigation, writing – original draft, writing – review and editing. **Duong Thi Hai Yen**: formal analysis, writing – original draft, data curation. **Do Thi Thao**: data curation, formal analysis, writing – review and editing. **Bui Huu Tai**: writing – original draft, writing – review and editing, data curation, formal analysis. **Nguyen Tuan Duong**: conceptualization, writing – original draft, writing – review and editing, methodology, data curation, investigation.

## Conflicts of Interest

The authors declare no conflicts of interest.

## Supporting information




Supporting Information for this article is available on the WWW under https://doi.org/10.1002/4332529. Additional references are cited within the Supporting Information [13, 14].
**Supporting file**: cbdv71687‐sup‐0001‐SuppMat.pdf

## Data Availability

The data that support the findings of this study are available in the Supporting Information of this article.

## References

[1] X. Geng , J. Wang , Y. W. Liu , et al., “Research Progress on Chemical Diversity of Saponins in *Panax Ginseng* ,” Chinese Herbal Medicines 16 (2024): 529–547. 39606259 10.1016/j.chmed.2024.08.005 PMC11589341

[2] B. K. Shin , S. W. Kwon , and J. H. Park , “Chemical Diversity of Ginseng Saponins From *Panax Ginseng* ,” Journal of Ginseng Research 39 (2015): 287–298, 10.1016/j.jgr.2014.12.005. 26869820 PMC4593792

[3] Y.‐Y. Lou , X. Zheng , Y.‐P. Huang , et al., “New Dammarane‐Type Triterpenoid Saponins From *Gynostemma pentaphyllum* and Their Sirt1 Agonist Activity,” Bioorganic Chemistry 116 (2012): e105357, 10.1016/j.bioorg.2021.105357. 34562675

[4] H. Z. Liang , P. X. Lu , L. L. Chu , et al., “Dammarane‐Type Saponins From *Gynostemma pentaphyllum* and Their Anti‐Aging Activities via Up‐Regulating Mitochondria Related Proteins,” Phytochemistry 213 (2023): e113744, 10.1016/j.phytochem.2023.113744. 37301356

[5] X. Q. Hu , W. L. Wang , D. M. Zhang , et al., “Phytochemistry, Bioactivities and Application of *Gynostemma pentaphyllum* Polysaccharide: A Review,” International Journal of Biological Macromolecules 322 (2025): e144964, 10.1016/j.ijbiomac.2025.144964. 40480562

[6] V. V. Chi , “Dictionary of Vietnamese Medicinal Plants,” Medical Publishing House 1 (2012): 609–670.

[7] X. J. Li , L. Liu , and S. Wei , “ *Gynostemma Pentaphyllum*: A Review on Its Traditional Uses, Phytochemistry and Pharmacology,” Journal of Functional Foods 124 (2025): e106651, 10.1016/j.jff.2024.106651.

[8] C. Su , N. Li , R. Ren , et al., “Progress in the Medicinal Value, Bioactive Compounds, and Pharmacological Activities of *Gynostemma pentaphyllum* ,” Molecules 26 (2021): e6249, 10.3390/molecules26206249. PMC8540791 34684830

[9] X. Li , Y. Chen , R. Wang , et al., “Gypenosides, a Promising Phytochemical Triterpenoid: Research Progress on Its Pharmacological Activity and Mechanism,” Frontiers in Pharmacology 16 (2025): e1705946, 10.3389/fphar.2025.1705946. PMC12571879 41181612

[10] V. Razmovski‐Naumovski , T. H. W. Huang , V. H. Tran , G. Q. Li , C. C. Duke , and B. D. Roufogalis , “Chemistry and Pharmacology of *Gynostemma pentaphyllum* ,” Phytochemistry Reviews 4 (2005): 197–219, 10.1007/s11101-005-3754-4.

[11] T. Takemoto , S. Arihara , and K. Yoshikawa , “Studies on the Constituents of Cucurbitaceae Plants. XIV,” Yakugaku Zasshi 106 (1986): 664–670, 10.1248/yakushi1947.106.8_664. 6470933

[12] L. Q. Cheng , J. R. Na , M. H. Bang , M. K. Kim , and D. C. Yang , “Conversion of Major Ginsenoside Rb1 to 20(S)‐ginsenoside Rg3 by Microbacterium sp. GS514,” Phytochemistry 69 (2008): 218–224, 10.1016/j.phytochem.2007.06.035. 17764709

[13] L. Voutquenne‐Nazabadioko , R. Gevrenova , N. Borie , et al., “Triterpenoid Saponins From the Roots of *Gypsophila Trichotoma* Wender,” Phytochemistry 90 (2013): 114–127, 10.1016/j.phytochem.2013.03.001. 23561300

[14] N. Van Quoc , B. Huu Tai , P. Hai Yen , et al., “Three Undescribed Furanoditerpenoids From the Tinospora Crispa That Inhibit no Production,” Chemistry & Biodiversity 21 (2025): e202401679, 10.1002/cbdv.202401679. 39136410

[15] L. H. Hu , Z. L. Chen , and Y. Y. Xie , “New Triterpenoid Saponins From Gynostemmapentaphyllum,” Journal of Natural Products 59 (1996): 1143–1145, 10.1021/np960445u. 8988599

[16] R. Teng , C. Ang , D. McManus , D. Armstrong , S. Mau , and A. Bacic , “Regioselective Acylation of Ginsenosides by Novozyme 435 to Generate Molecular Diversity,” Helvetica Chimica Acta 87 (2004): 1860–1872, 10.1002/hlca.200490165.

[17] C. Lee , J. W. Lee , Q. H. Jin , et al., “Isolation and Characterization of Dammarane‐Type Saponins From Gynostemma Pentaphyllum and Their Inhibitory Effects on IL‐6‐Induced STAT3 Activation,” Journal of Natural Products 78 (2015): 971–976, 10.1021/np500803e. 25895106

[18] H. Liu , S. F. Xing , W. Y. Cui , M. L. Zu , C. L. Lyu , and X. L. Piao , “A Novel Dammarane‐type Saponin From *Gynostemma Pentaphyllum* and Its Neuroprotective Effect,” China Journal of Chinese Materia Medica 2 (2021): 380–387. 10.19540/j.cnki.cjcmm.20200807.201 33645125

[19] B. H. Tai , N. M. Cuong , T. T. Huong , E. M. Choi , A. J. Kim , and Y. H. Kim , “Chrysoeriol Isolated From the Leaves of Eurya Ciliata Stimulates Proliferation and Differentiation of Osteoblastic MC3T3‐E1 Cells,” Journal of Asian Natural Products Research 11 (2009): 817–823, 10.1080/10286020903117317. 20183330

[20] E. H. Alcantara , M. Y. Shin , H. Y. Sohn , et al., “Diosgenin Stimulates Osteogenic Activity by Increasing Bone Matrix Protein Synthesis and Bone‐Specific Transcription Factor Runx2 in Osteoblastic MC3T3‐E1 Cells,” Journal of Nutritional Biochemistry 22 (2011): 1055–1063, 10.1016/j.jnutbio.2010.09.003. 21292464

[21] Y. Ding , C. Liang , H. T. Nguyen , E. M. Choi , J. A. Kim , and Y. H. Kim , “Chemical Constituents From *Acer Mandshuricum* and Their Effects on the Function of Osteoblastic MC3T3‐E1 Cells,” Bulletin of the Korean Chemical Society 31 (2010): 929–933, 10.5012/bkcs.2010.31.04.929.

[22] L. D. Quarles , D. A. Yohay , L. W. Lever , R. Caton , and R. J. Wenstrup , “Distinct Proliferative and Differentiated Stages of Murine MC3T3‐E1 Cells in Culture: An *In Vitro* Model of Osteoblast Development,” Journal of Bone and Mineral Research 7 (1992): 683–692, 10.1002/jbmr.5650070613. 1414487

[23] S. Ansari , K. Ito , and S. Hofmann , “Alkaline Phosphatase Activity of Serum Affects Osteogenic Differentiation Cultures,” ACS Omega 7 (2022): e12724, 10.1021/acsomega.1c07225. PMC9026015 35474849

[24] R. Liu , L. X. Xu , L. J. Tong , et al., “Therapeutic Effects of Ginsenosides on Osteoporosis for Novel Drug Applications,” European Journal of Pharmacology 974 (2024): e176604, 10.1016/j.ejphar.2024.176604. 38649090

[25] C. Inderjeeth and D. C. Inderjeeth , “Novel Therapies in Osteoporosis—Clinical Update – 2025,” Best Practice & Research Clinical Rheumatology 39 (2025): e102100, 10.1016/j.berh.2025.102100. 40998596

