# Supplementary material for: Osteogenic Activity of New Dammarane‐Type Saponins From Gynostemma pentaphyllum
Source: Chem Biodivers. 2026 Sep 10;23(9):e71687. doi: 10.1002/cbdv.71687 (PMC13559854; doi:10.1002/cbdv.71687)
Supplement: Supplementary file 1 [file CBDV-23-e71687-s001.pdf]

Content

**Experimental**		2
General		2
Extraction and isolation		2
Acid hydrolysis of compounds 1 and 2		3
Figure S1	HR-ESI-MS of compound **1**	4
Figure S2	^{1}H NMR spectra of compound **1**	5
Figure S3	Expanded ^{1}H NMR spectra of compound **1**	6
Figure S4	^{13}C NMR spectra of compound **1**	7
Figure S5	HSQC spectra of compound **1**	8
Figure S6	HMBC spectra of compound **1**	9
Figure S7	Expanded HMBC spectra of compound **1**	10
Figure S8	COSY spectrum of compound **1**	11
Figure S9	NOESY spectrum of compound **1**	12
Figure S10	HR-ESI-MS of compound **2**	13
Figure S11	^{1}H NMR spectra of compound **2**	14
Figure S12	Expanded ^{1}H NMR spectra of compound **2**	15
Figure S13	^{13}C NMR spectra of compound **2**	16
Figure S14	HSQC spectra of compound **2**	17
Figure S15	HMBC spectra of compound **2**	18
Figure S16	Expanded HMBC spectra of compound **1**	19
Figure S17	COSY spectrum of compound **2**	20
Figure S18	NOESY spectrum of compound **2**	21
Figure S19	^{1}H NMR spectra of compound **3**	22
Figure S20	Expanded ^{1}H NMR spectra of compound **3**	23
Figure S21	^{13}C NMR spectra of compound **3**	24
Figure S22	HSQC spectrum of compound **3**	25
Figure S23	HMBC spectrum of compound **3**	25
Figure S24	^{1}H NMR spectra of compound **4**	26
Figure S25	^{13}C NMR spectrum of compound **4**	27
Figure S26	HSQC spectra of compound **4**	27
Figure S27	HMBC spectra of compound **4**	28
Figure S28	^{1}H NMR spectra of compound **5**	29
Figure S29	Expanded ^{1}H NMR spectra of compound **5**	30
Figure S30	^{13}C NMR spectrum of compound **5**	31
Figure S31	Effects of compounds **1-5** on the viability of MC3T3-E1 cells.	32
Table S1	NMR data of compound **3**	33
Table S2	NMR data of compound **4**	34
Table S3	NMR data of compound **5**	35

Experimental

General

The optical rotations were measured on a Jasco P2000-polarimeter. The HR-ESI-MS was measured on an Agilent 6530 Accurate Mass Q-TOF LC/MS. The HR-ESI-MS was measured on an Agilent 6530 Accurate Mass Q-TOF LC/MS. The NMR spectra were recorded on a Bruker 600 or 500 MHz spectrometer. The preparative HPLC were run on an Agilent-1100 system including quaternary pump, DAD detector, autosampler, and preparative HPLC column YMC J'sphere ODS-H80 (4 µm, 20 × 250 mm). Isocratic mobile phase with the flow rate of 3 mL/min was used in pre-HPLC. The compound was monitored at wavelengths of 205, 230, 254, and 280 nm. Flash column chromatography was run using silica gel, reversed phase C-18, and diaion HP-20 resins as stationary phase. Thin layer chromatography was carried out on pre-coated silica gel 60 F_{254} and RP-18 F_{254S} plates. The spots were detected by spraying with aqueous solution of H_2SO_4 5% followed by heating with a heat gun.

Extraction and isolation

The dried and powdered aerial parts of *G. pentaphyllum* (3.0 kg) were sonicated in MeOH three times to yield 280 g MeOH extract. This was suspended in water and successively partitioned with *n*-hexane, dichloromethane, then ethyl acetate to afford the corresponding extracts (GP1, 27.0 g), GP2 (35 g), GP3 (28 g), and the remaining aqueous fraction (GP4). Because *G. pentaphyllum* tea is traditionally consumed as a water decoction and, according to published literature, the major constituents of this plant are saponins, the aqueous extract was selected for this study. This fraction was subjected to chromatography on a Diaion HP-20 column, eluting with MeOH in water (25, 50, 75, and 100% MeOH) to afford four fractions, GP4A–GP4D. Based on TLC analysis, fraction GP4B (23 g) was further separated by silica gel column chromatography using CH_2Cl_2/MeOH (20:1, 7:1, and 1:1; 1.5 L each) as the eluent, yielding three fractions, GP4B1–GP4B3. Fraction GP4B2 (3.8 g) was further chromatographed on a YMC RP-18 column, eluting with MeOH/H_2O (1:1.2, 3 L), to afford four fractions, GP4B2A–GP4B2D. Fraction GP4B2B (450 mg) was chromatographed on an HPLC using J'sphere ODS H-80, 250mm×20mm column, ACN 20% in H_2O (flow rate of 3 mL/min) to yield compounds **1** (13 mg, t_R 47.2 min) and **5** (18.6 mg, t_R 51.4 min). Fraction GP4B2C (345 mg) was chromatographed on an HPLC using 22% ACN to get compounds **2** (13.4 mg, t_R 50.2 min) and **3** (15.2 mg, t_R 56.7 min). Fraction GP4B2D was chromatographed on an HPLC using 25% ACN to get compound **4** (15.3 mg, t_R 49.4 min).

Acid hydrolysis of compounds 1 and 2

Compounds **1** and **2** (each 4.0 mg) was separately dissolved in 1.0 M HCl (dioxane−H_2O, 1:1, v/v, 1.0 mL) and heated to 80 °C in a water bath for 3 h. The acidic solution was neutralized with silver carbonate, the precipitated silver chloride was removed, and the solution was concentrated thoroughly under a nitrogen atmosphere. The residue was re-dissolved in 1.0 mL of water and extracted with chloroform (three times, each 1 mL). The aqueous layer was concentrated to dryness using nitrogen gas and monosaccharides were purified by preparative TLC (pre-coated silica gel 60 F_{254}, MeCOEt–isoPrOH–Me_2CO–H_2O (20:10:7:6)) to get glucose (R_f = 0.41, 1.4 mg) and xylose (R_f = 0.61, 0.6 mg) for **1**, glucose (R_f = 0.41, 1.2 mg) and xylose (R_f = 0.61, 0.7 mg) for **2**. The specific rotations $[\alpha]_D^{25}$ of sugars were determined after dissolving in H_2O; Compound **1**: found D-glucose: $[\alpha]_D^{25}$ +48.0 (*c* 0.1, H_2O) and D-xylose $[\alpha]_D^{25}$ +20.0 (*c* 0.06, H_2O). Compound **2**: D-glucose: found $[\alpha]_D^{25}$ +48.0 (*c* 0.08, H_2O) and D-xylose $[\alpha]_D^{25}$ +20.0 (*c* 0.07, H_2O) [10,11]

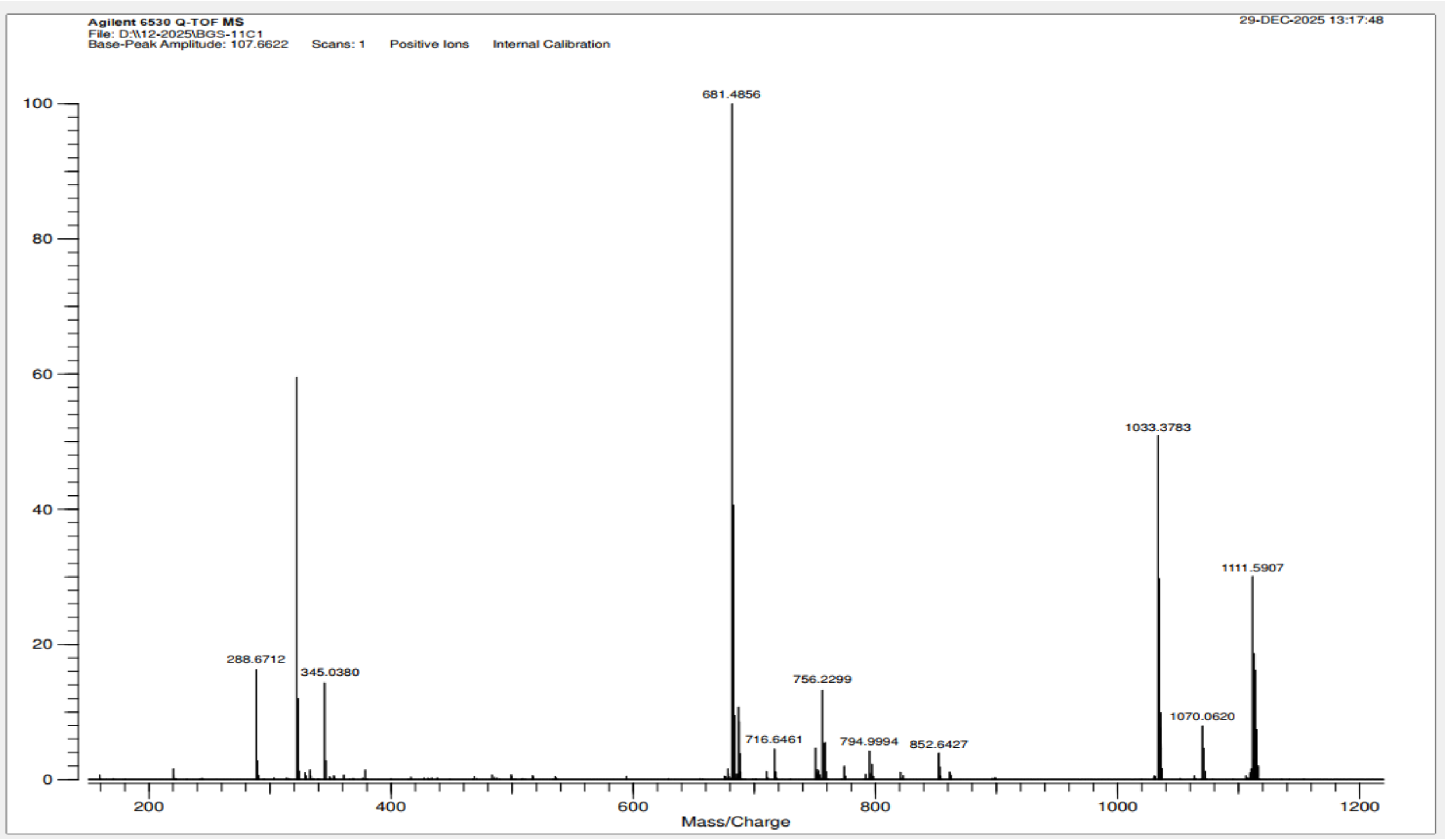

HRESIMS *m/z* 1111.5907 $[M+H]^+$ (calcd for $C_{53}H_{91}O_{24}^+$: 1111.5895, Δ= +1.1 ppm)

Elemental Composition Search Report:

Target Mass:

Target m/z = 1111.5907 ± 0.002

Charge = +1

Possible Elements:

Element:	Exact Mass:	Min:	Max:
C	12.000000	0	55
H	1.007825	0	100
N	14.003074	0	5
O	15.994915	0	25
S	31.972071	0	3

Additional Search Restrictions:

None

Search Results:

Number of Hits = 9

m/z	Delta m/z	DBE	Formula
1111.59082	-0.00012	13.5	$C_{54}H_{87}N_4O_{20}^{+1}$
1111.59086	-0.00016	-5.0	$C_{40}H_{97}N_5O_{25}S_2^{+1}$
1111.59035	0.00035	7.5	$C_{54}H_{95}O_{19}S_2^{+1}$
1111.59151	-0.00081	4.0	$C_{48}H_{93}N_3O_{23}S^{+1}$
1111.59169	-0.00099	12.5	$C_{55}H_{91}N_4O_{15}S_2^{+1}$
1111.58970	0.00100	-1.5	$C_{46}H_{99}N_2O_{21}S_3^{+1}$
1111.58949	0.00121	8.5	$C_{53}H_{91}O_{24}^{+1}$
1111.59238	-0.00168	3.0	$C_{49}H_{97}N_3O_{18}S_3^{+1}$
1111.58901	0.00169	8.0	$C_{52}H_{93}N_3O_{18}S_2^{+1}$

Figure S1. HR-ESI-MS of compound **1**

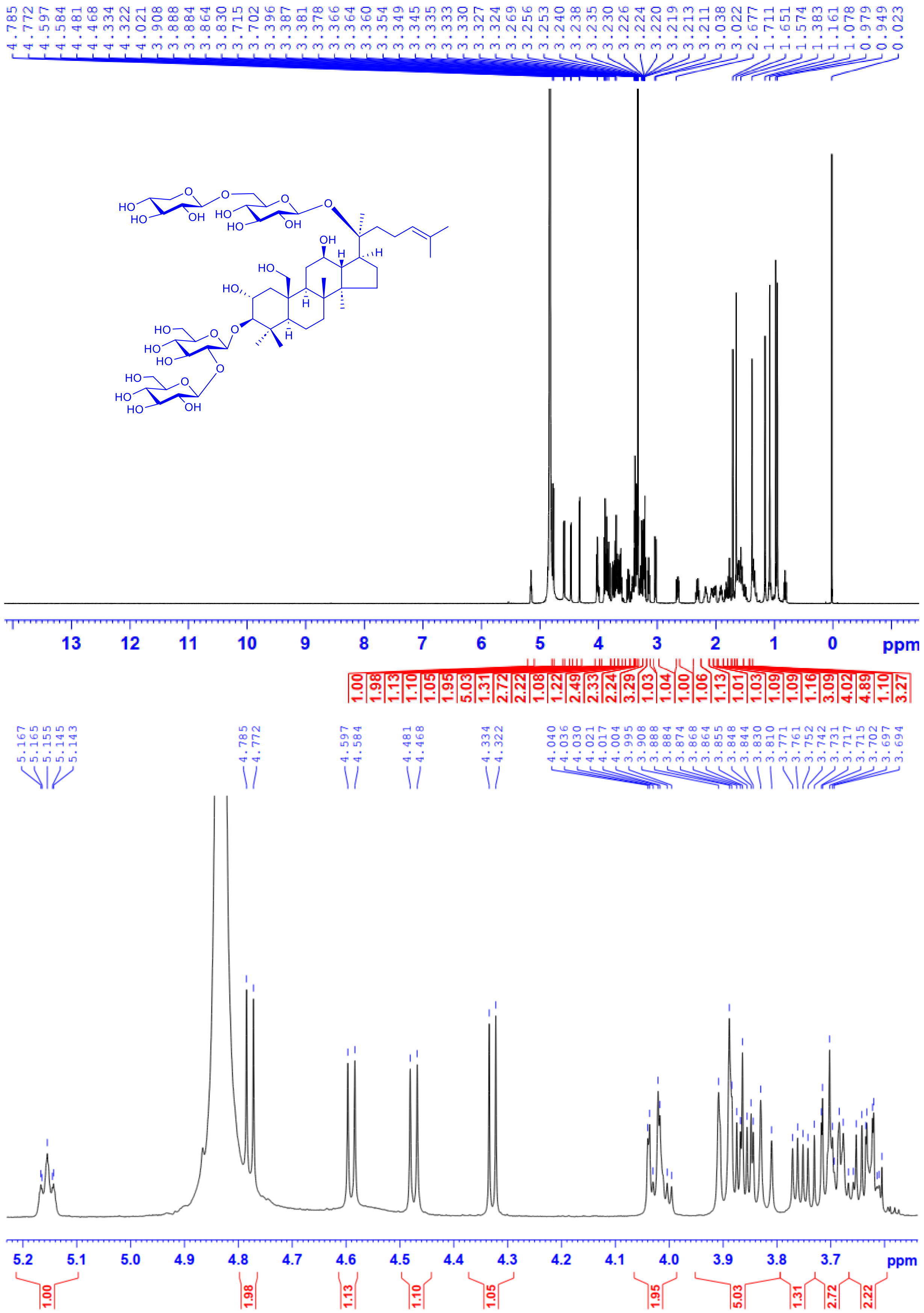

Figure S2. ^{1}H-NMR spectrum of compound **1**

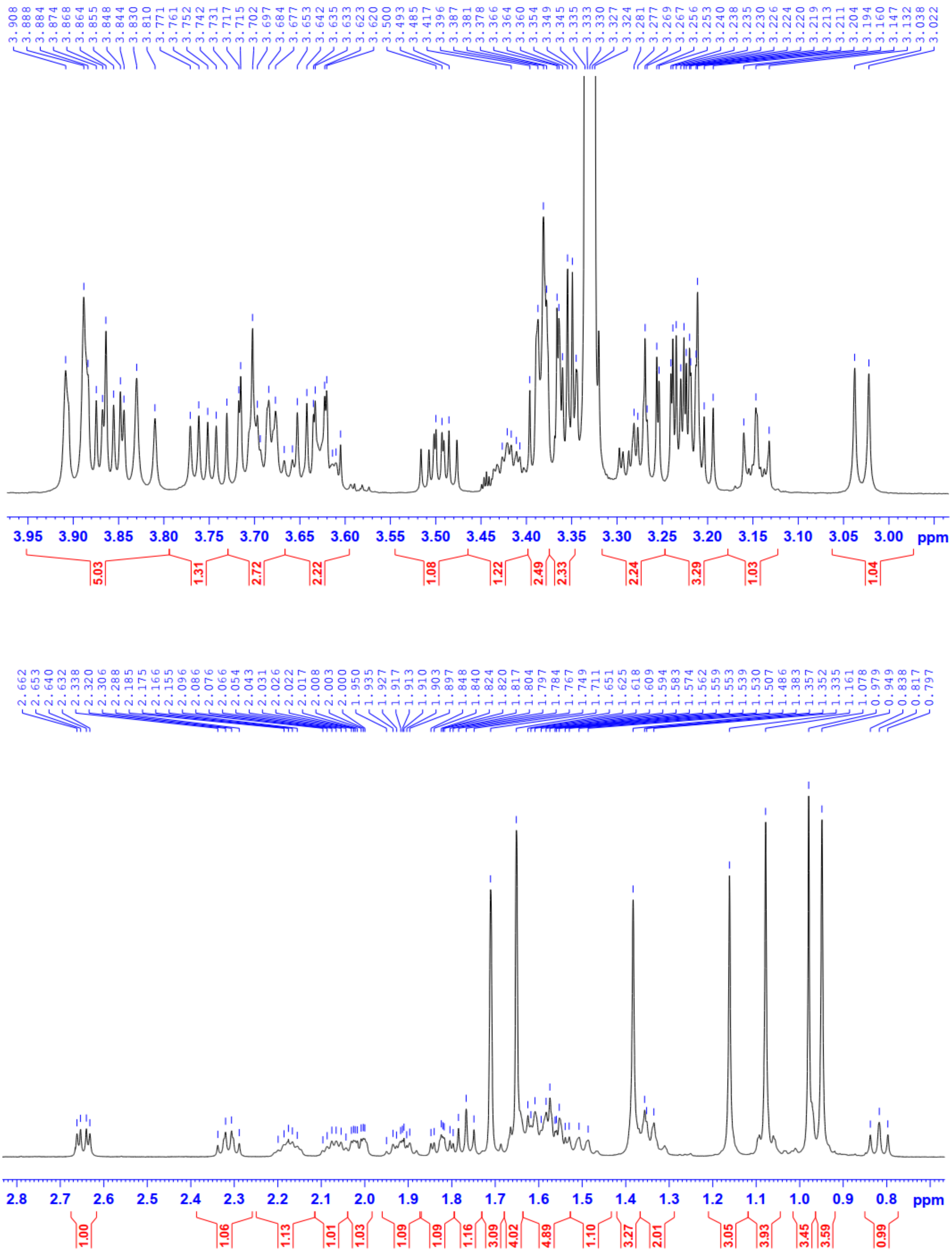

Figure S3. Expanded ^{1}H-NMR spectrum of compound **1**

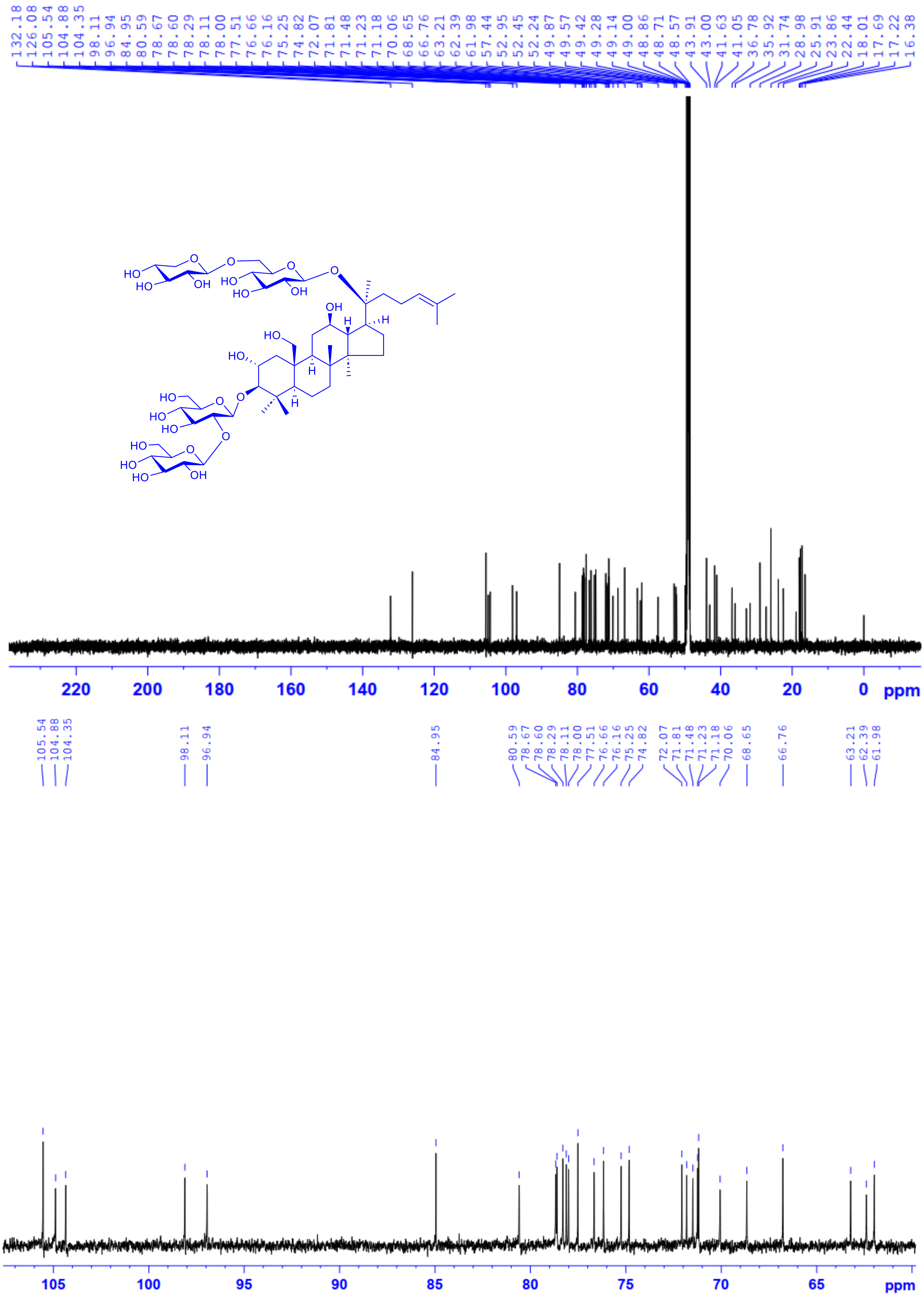

Figure S4. ^{13}C NMR spectrum of compound **1**

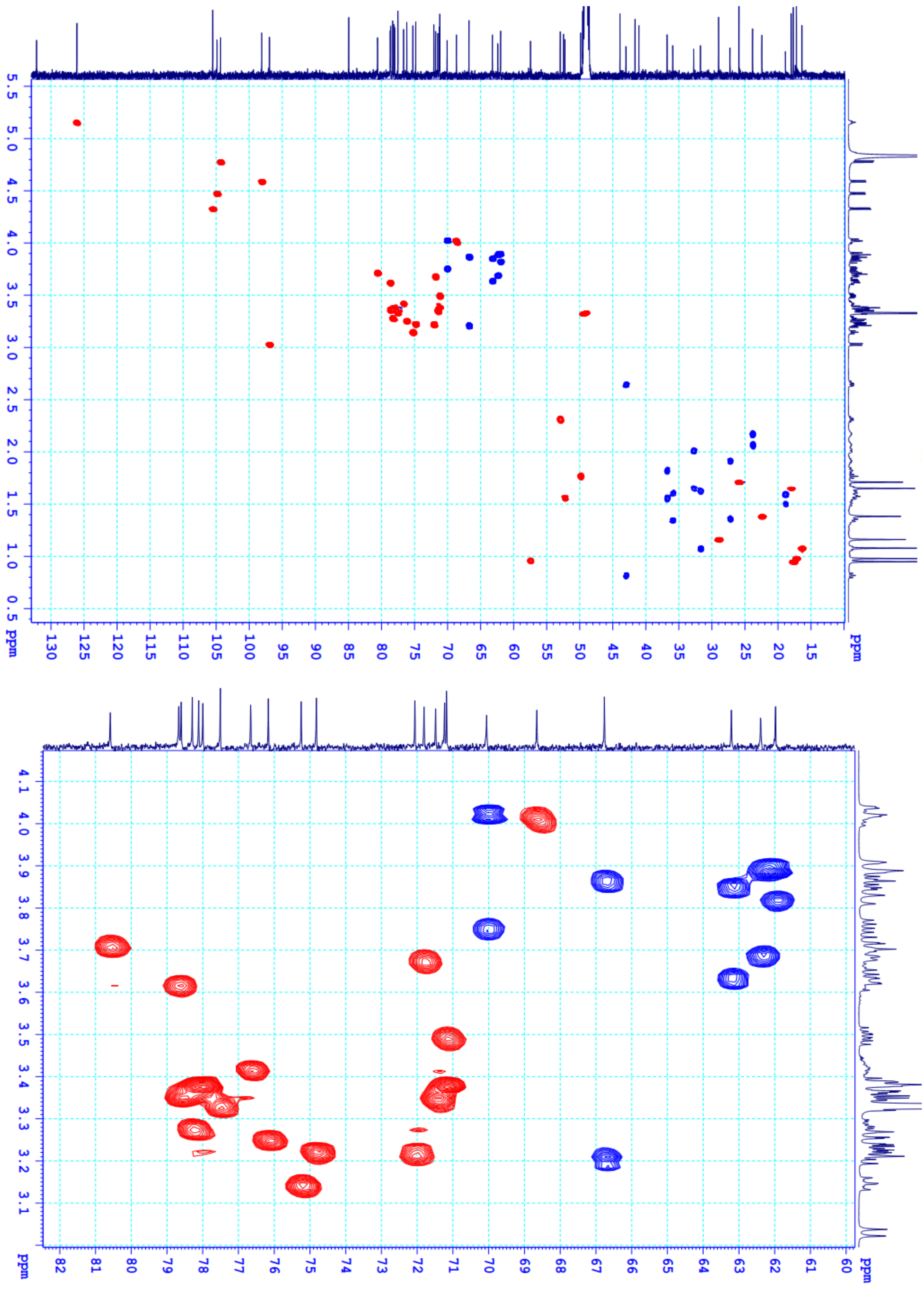

Figure S5. HSQC spectrum of compound **1**

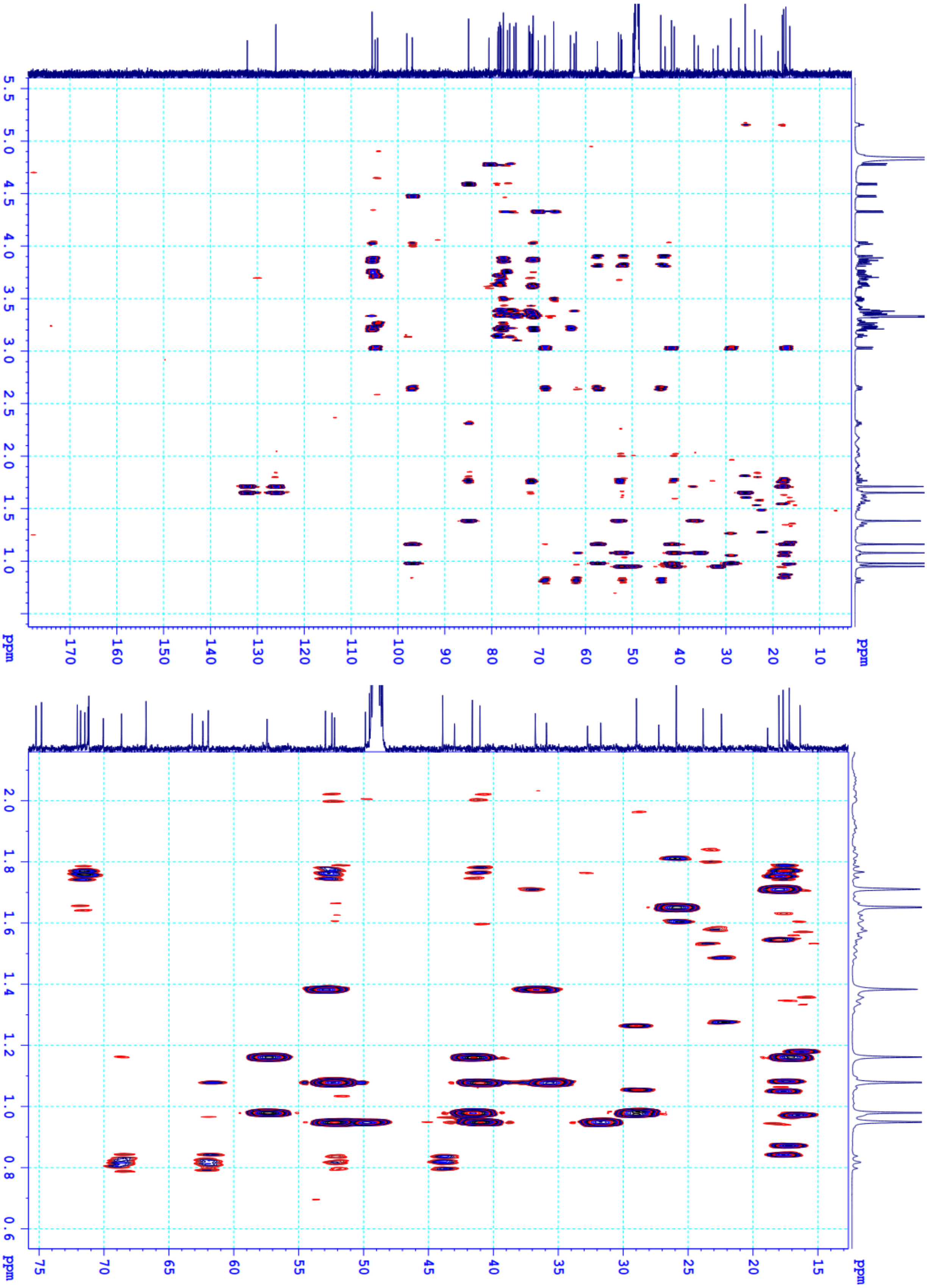

Figure S6. HMBC spectrum of compound **1**

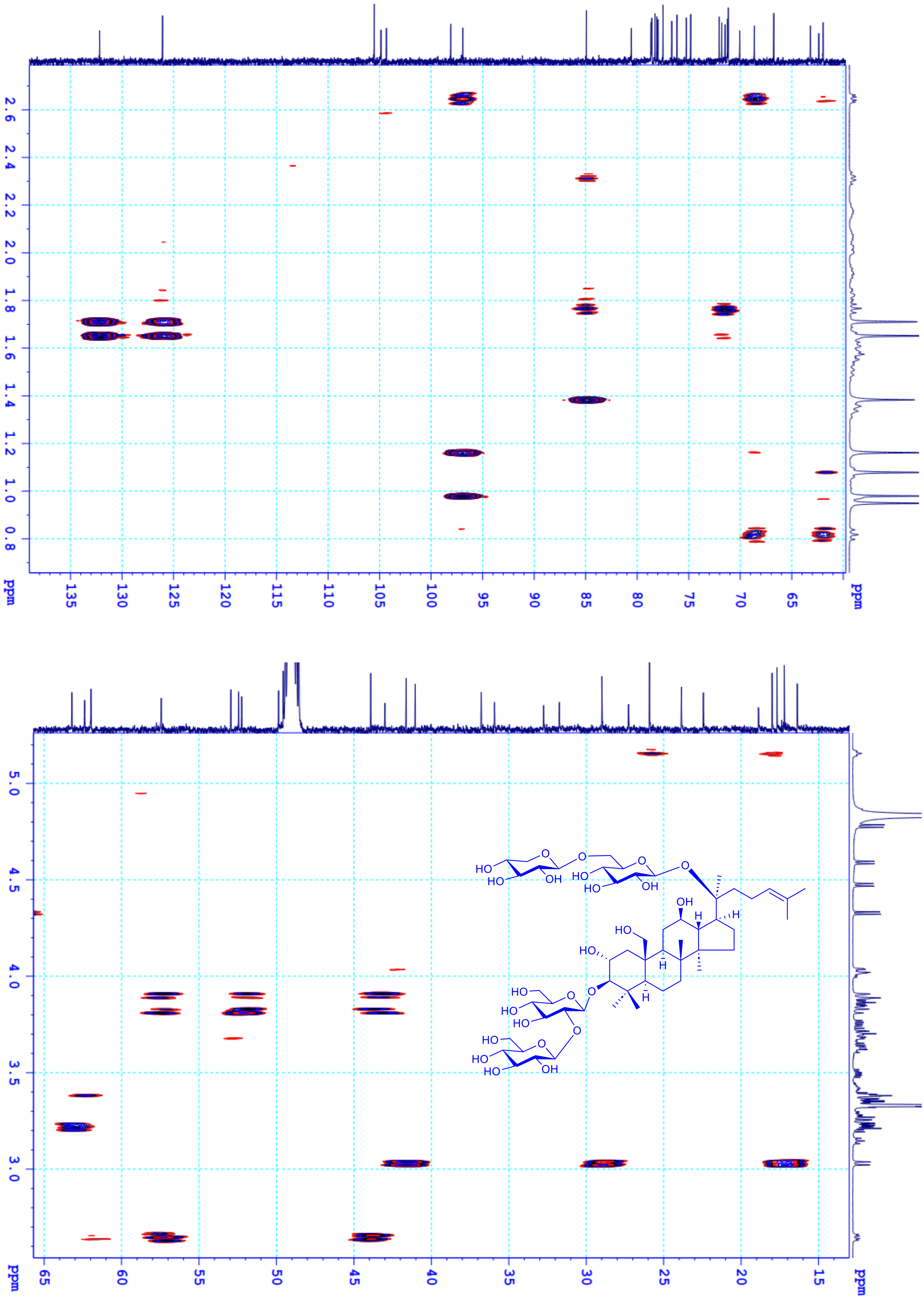

Figure S7. Expanded HMBC spectrum of compound **1**

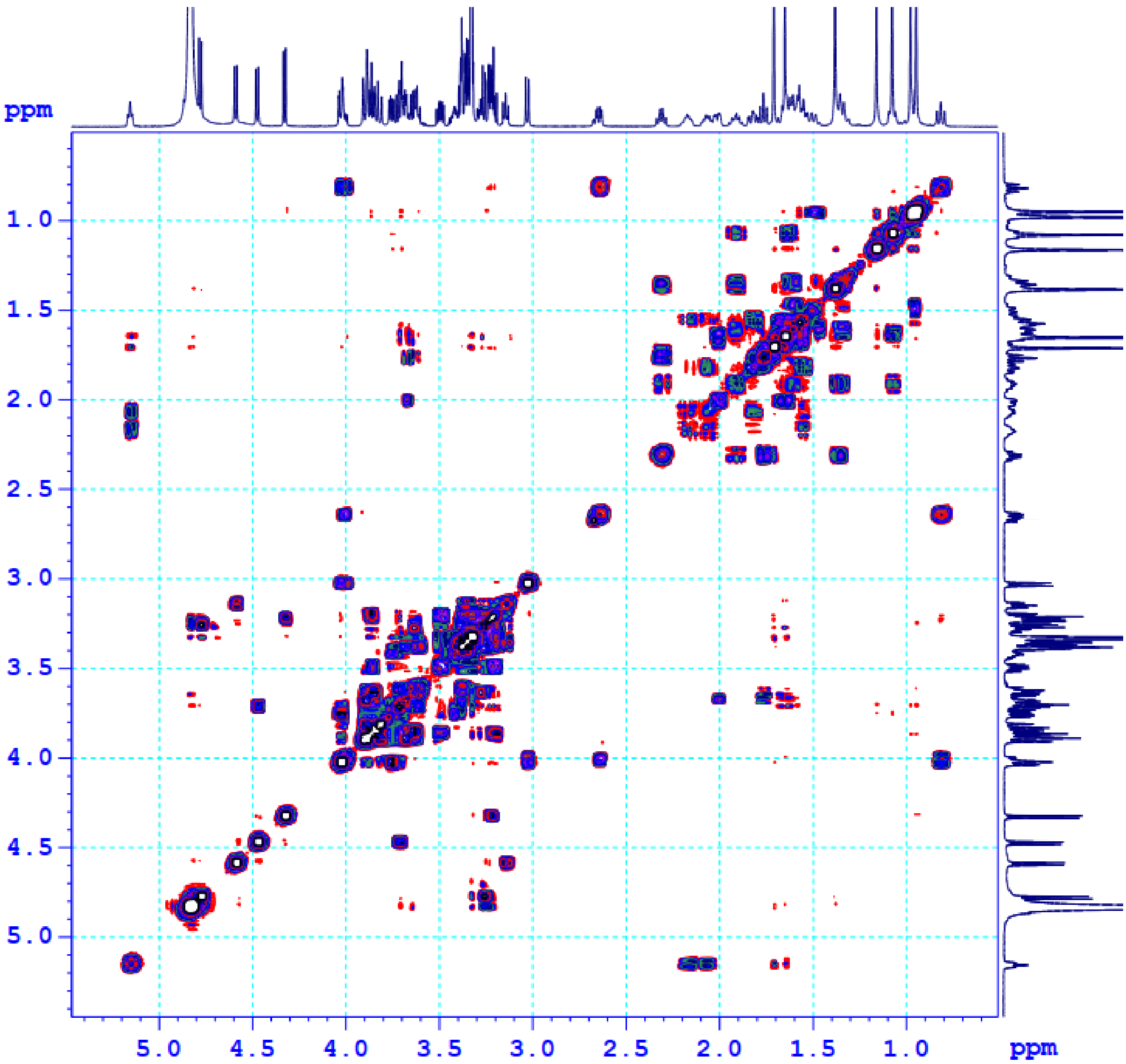

Figure S8. COSY spectrum of compound **1**

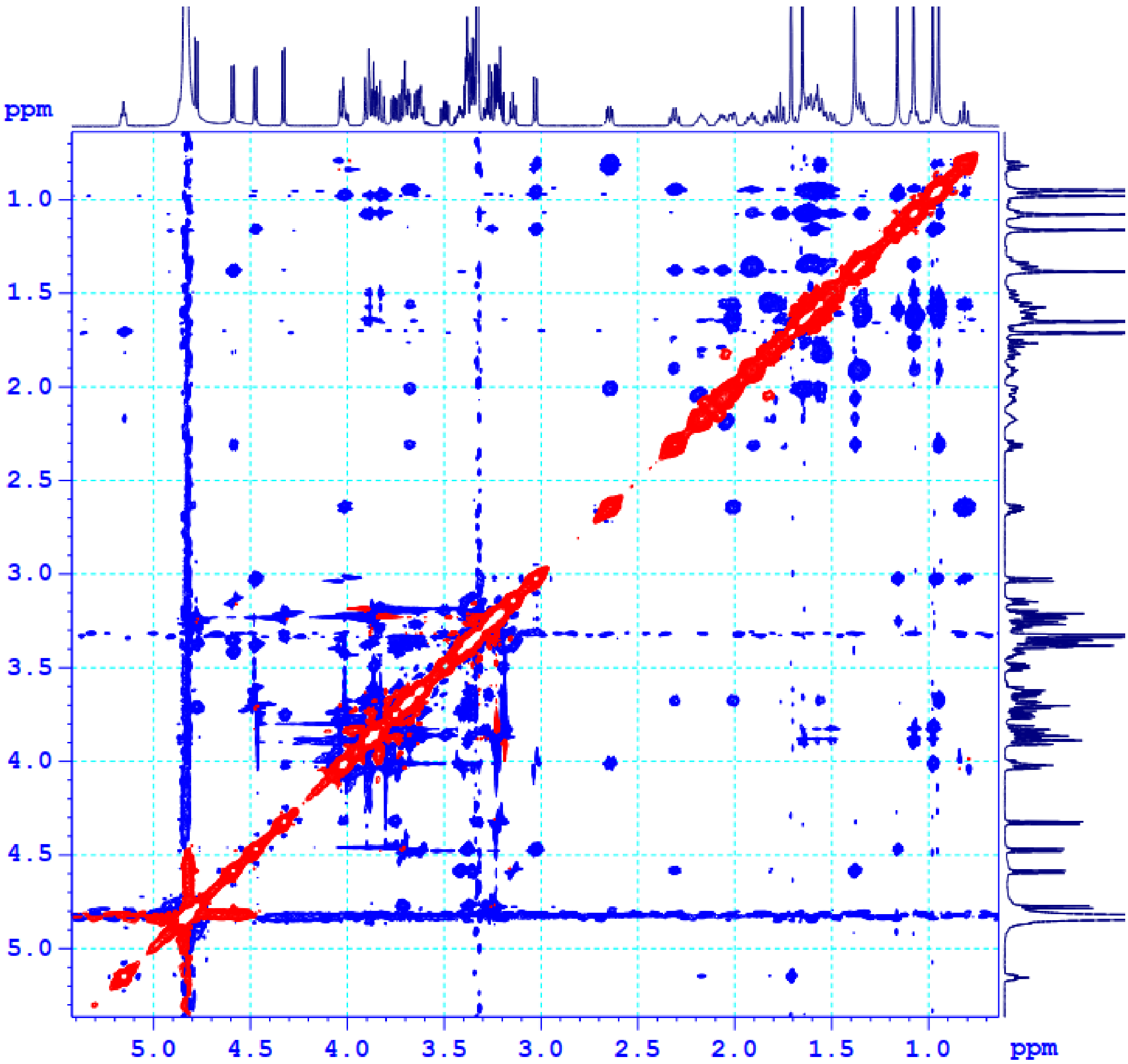

Figure S9. NOESY spectrum of compound **1**

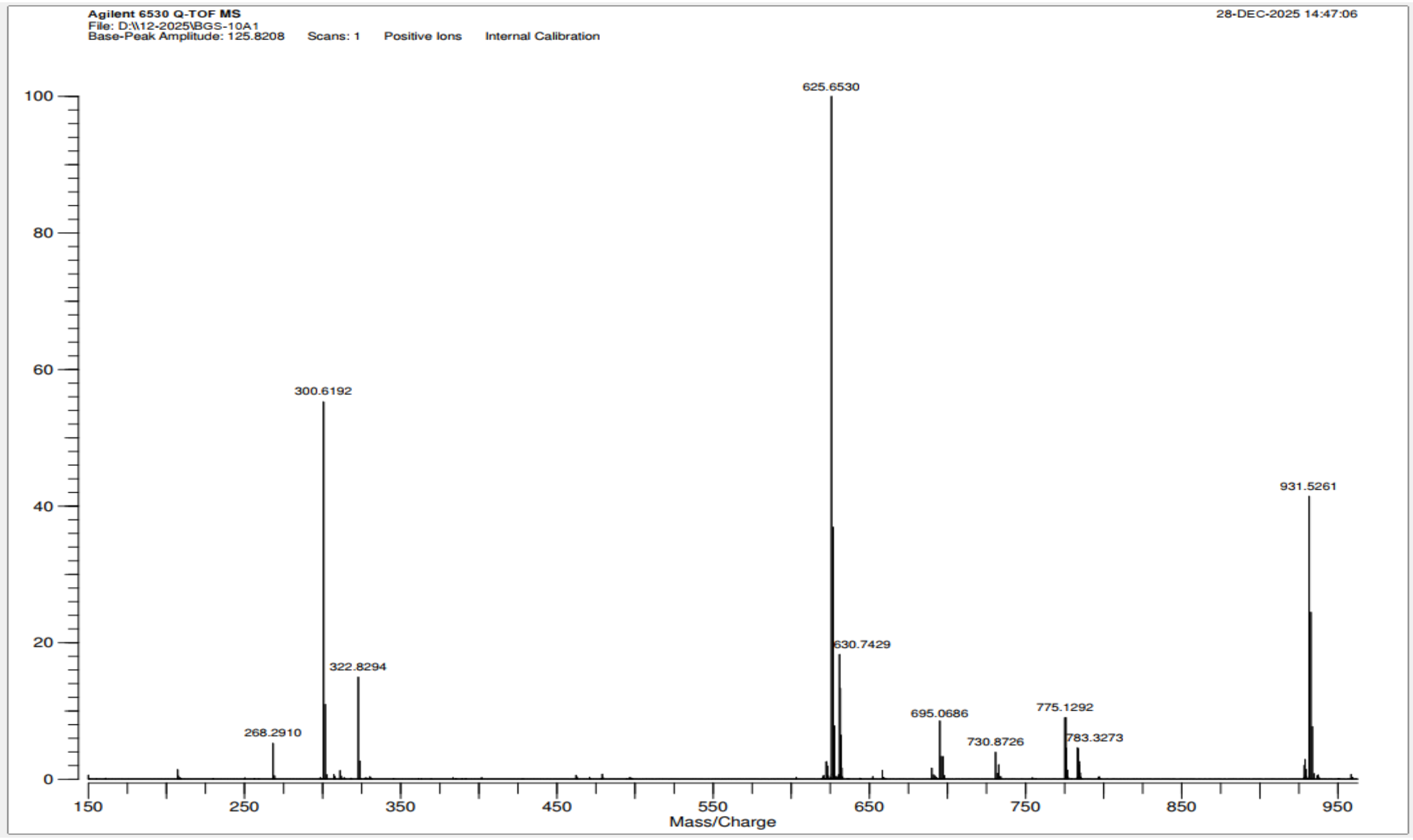

HRESIMS *m/z* 931.5261 [M+H]$^+$ (calcd for $C_{47}H_{79}O_{18}^+$: 931.5261, Δ= 0 ppm)

Elemental Composition Search Report:

Target Mass:

Target m/z = 931.5261 ± 0.002

Charge = +1

Possible Elements:

Element:	Exact Mass:	Min:	Max:
C	12.000000	0	50
H	1.007825	0	85
N	14.003074	0	5
O	15.994915	0	20
S	31.972071	0	3

Additional Search Restrictions:

None

Search Results:

Number of Hits = 9

m/z	Delta m/z	DBE	Formula
931.52610	0.00000	8.5	$C_{47}H_{79}O_{18}^{+1}$
931.52562	0.00048	8.0	$C_{46}H_{81}N_3O_{12}S_2^{+1}$
931.52545	0.00065	-0.5	$C_{39}H_{83}N_2O_{20}S^{+1}$
931.52696	-0.00086	7.5	$C_{48}H_{83}O_{13}S_2^{+1}$
931.52497	0.00113	-1.0	$C_{38}H_{85}N_5O_{14}S_3^{+1}$
931.52743	-0.00133	13.5	$C_{48}H_{75}N_4O_{14}^{+1}$
931.52475	0.00135	9.0	$C_{45}H_{77}N_3O_{17}^{+1}$
931.52747	-0.00137	-5.0	$C_{34}H_{85}N_5O_{19}S_2^{+1}$
931.52410	0.00200	0.0	$C_{37}H_{81}N_5O_{19}S^{+1}$

Figure S10. HR-ESI-MS of compound **2**

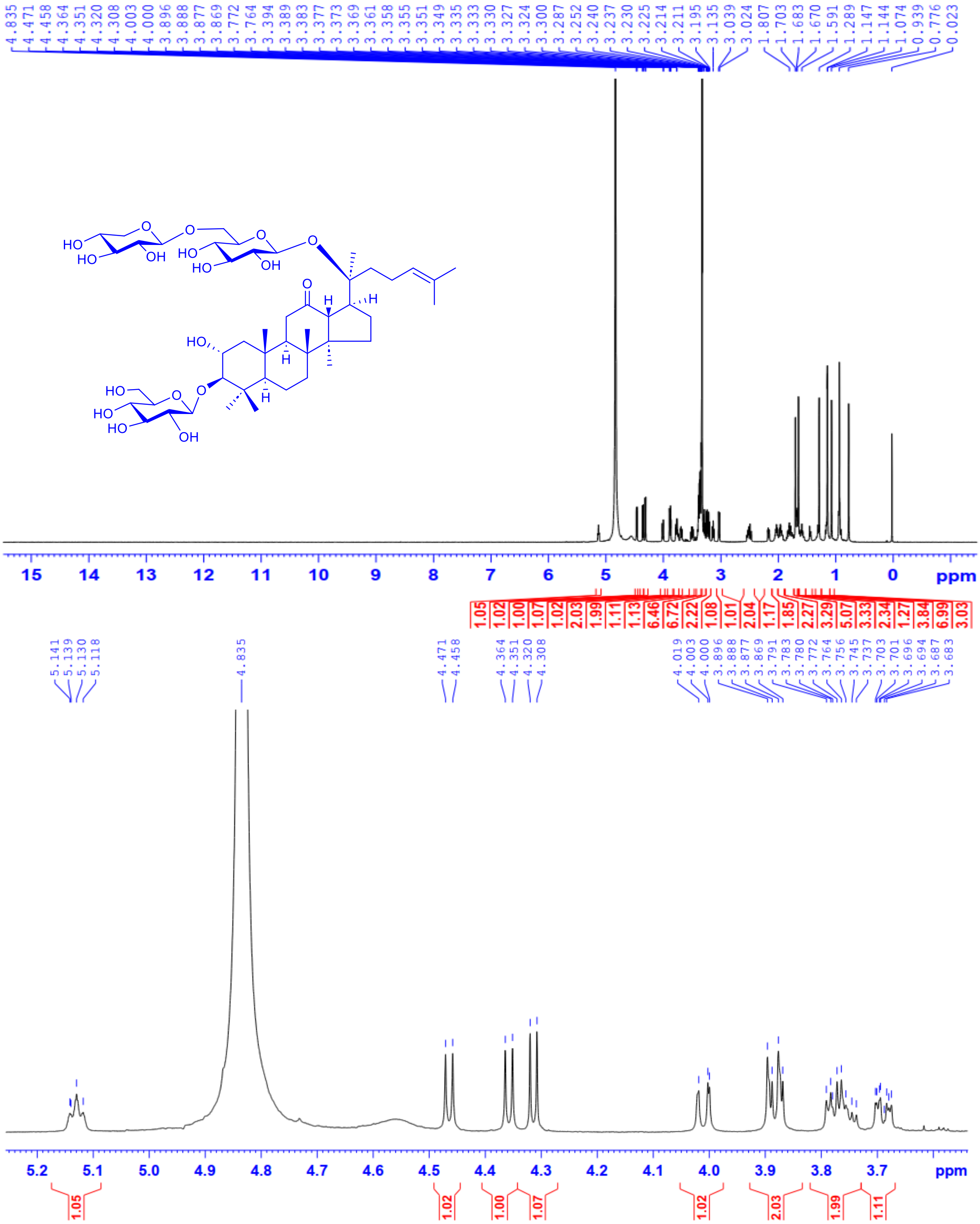

Figure S11. ^{1}H NMR spectra of compound **2**

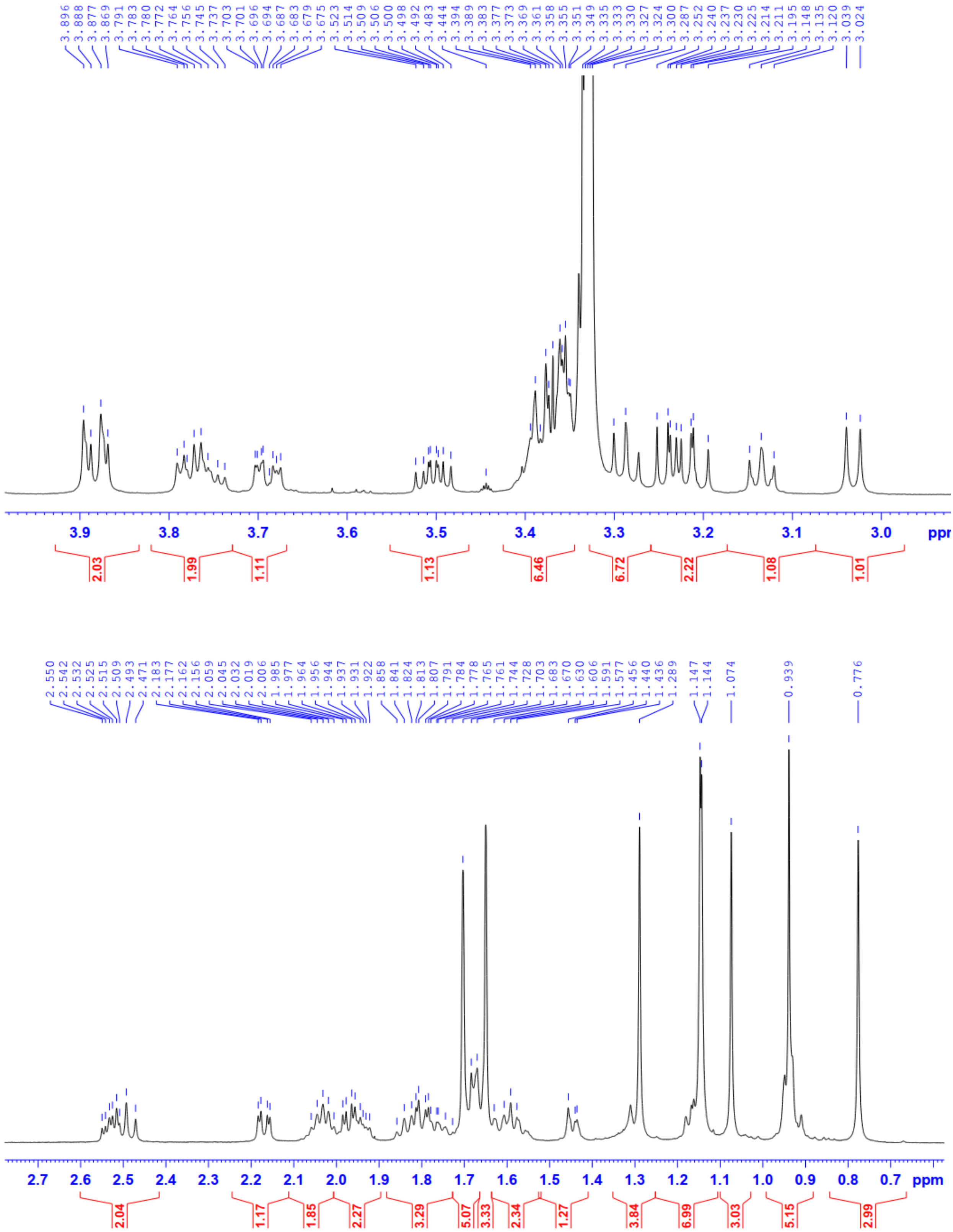

Figure S12. Expanded ^{1}H NMR spectra of compound **2**

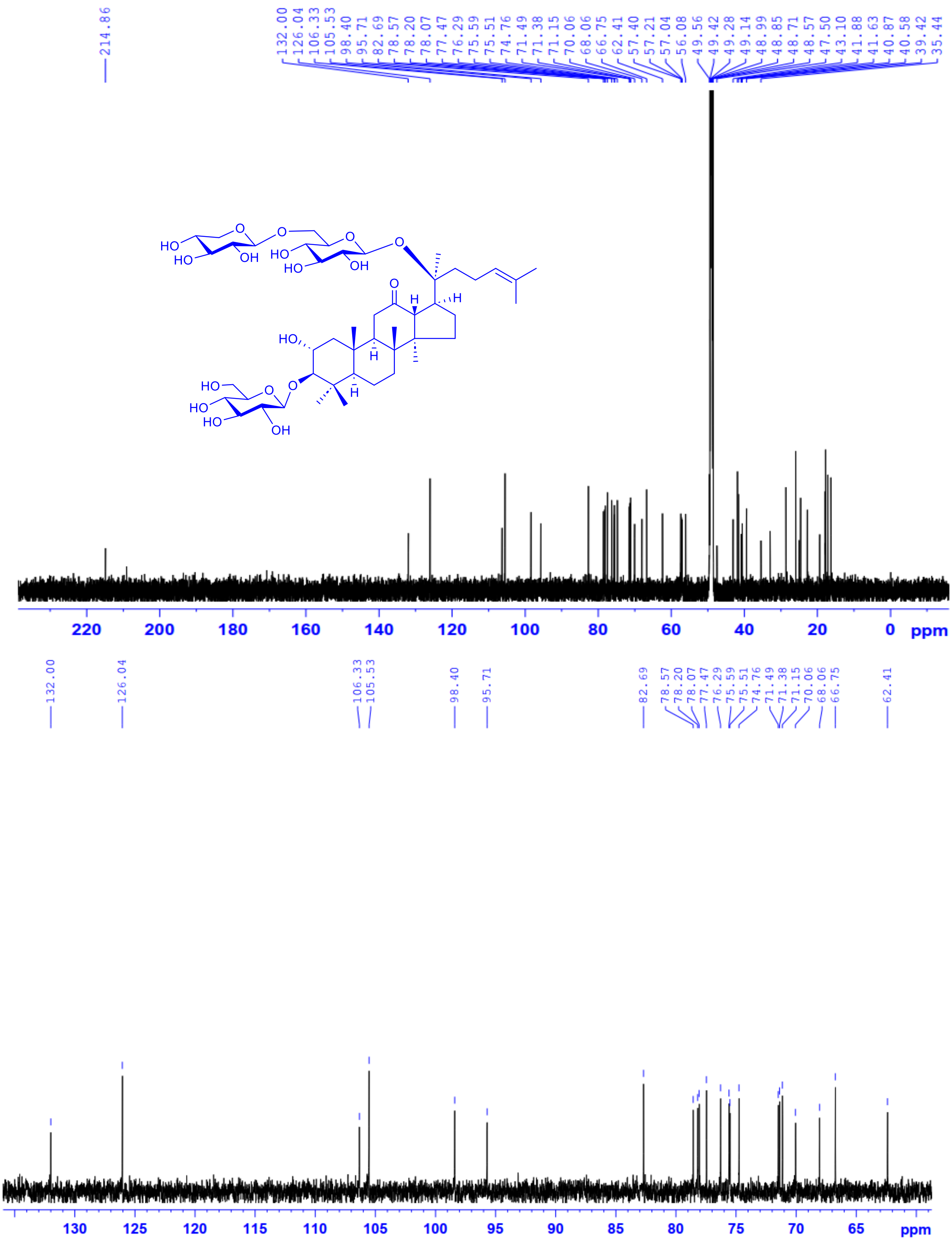

Figure S13. ^{13}C NMR spectrum of compound **2**

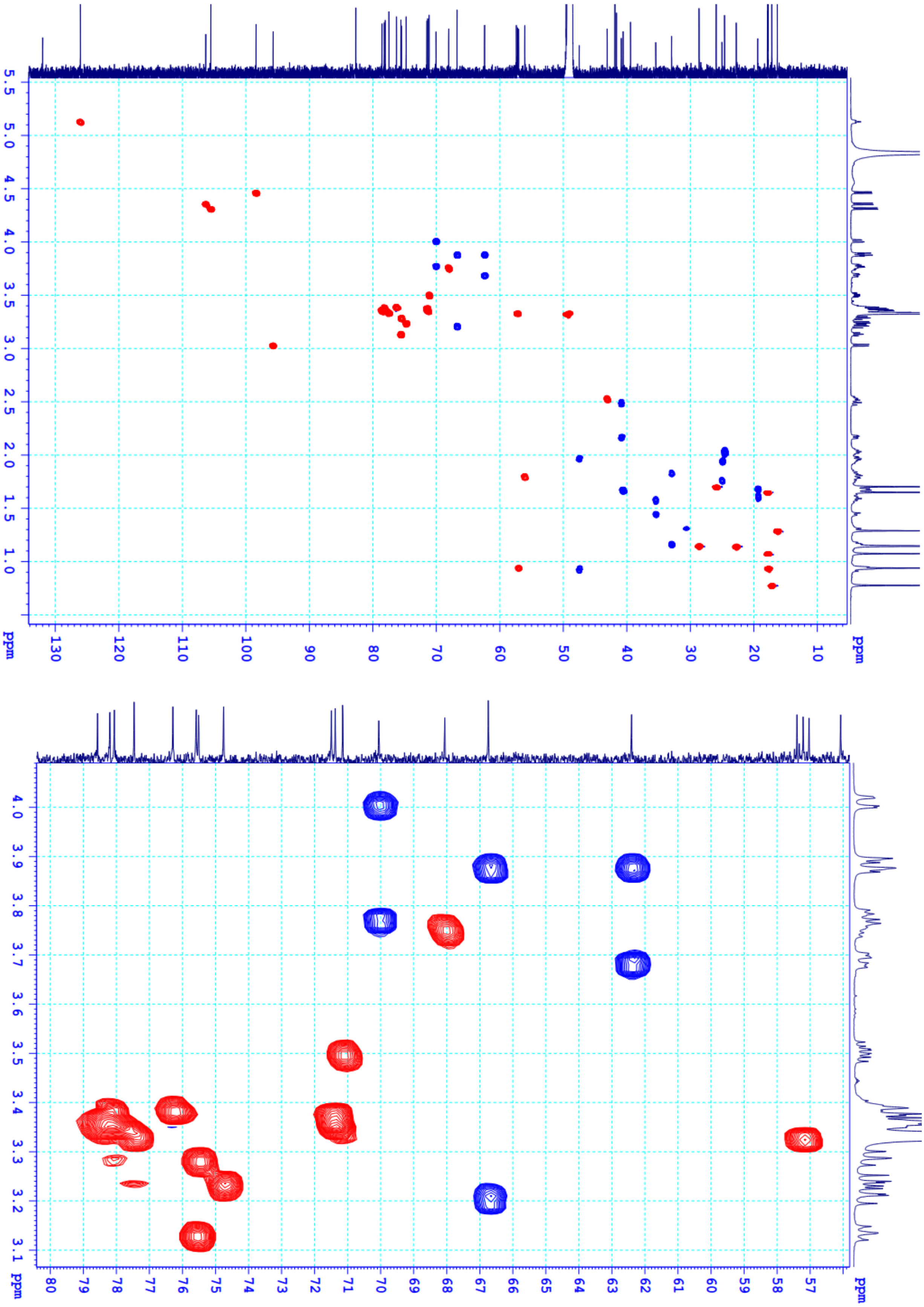

Figure S14. HSQC spectrum of compound **2**

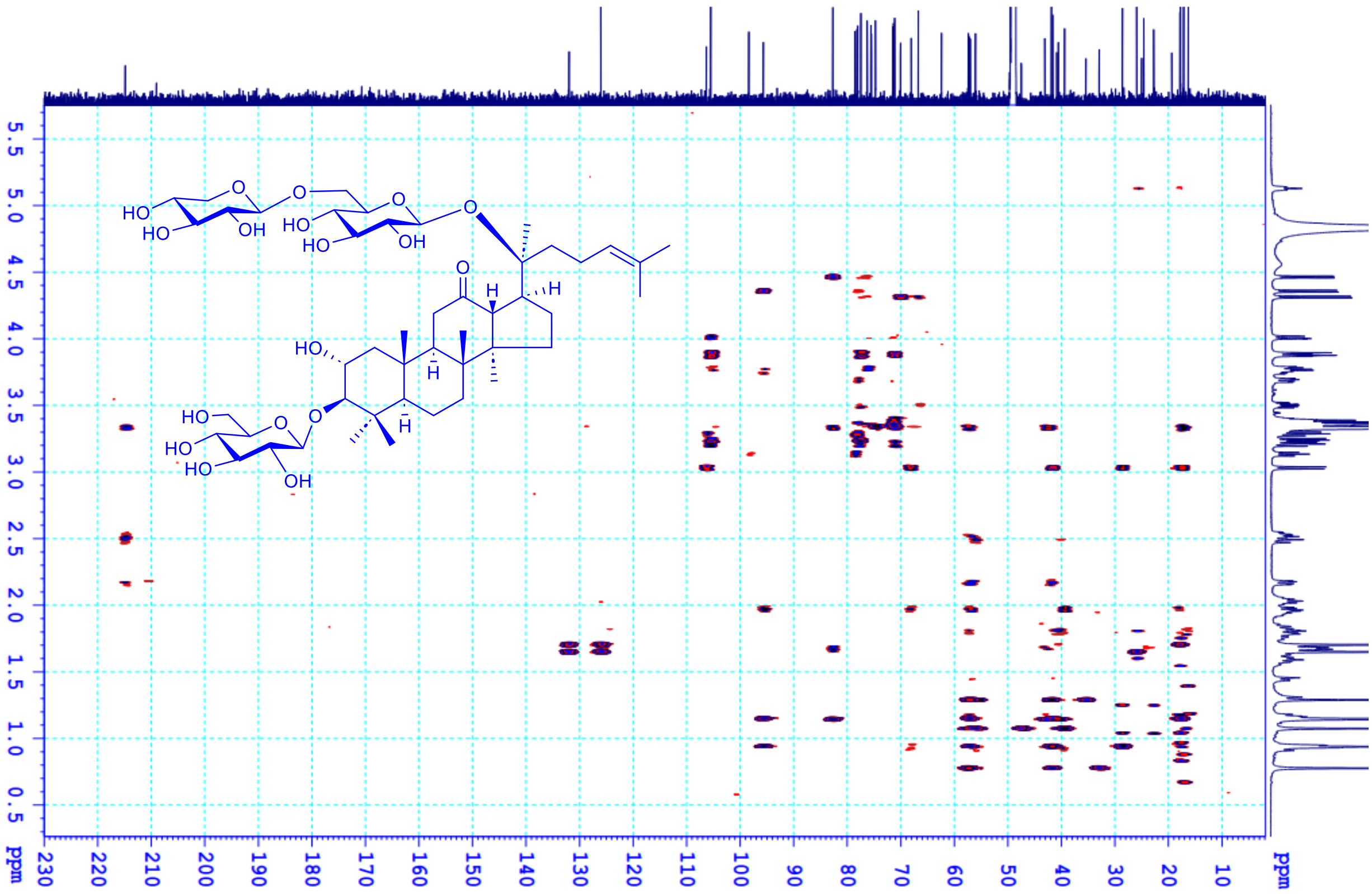

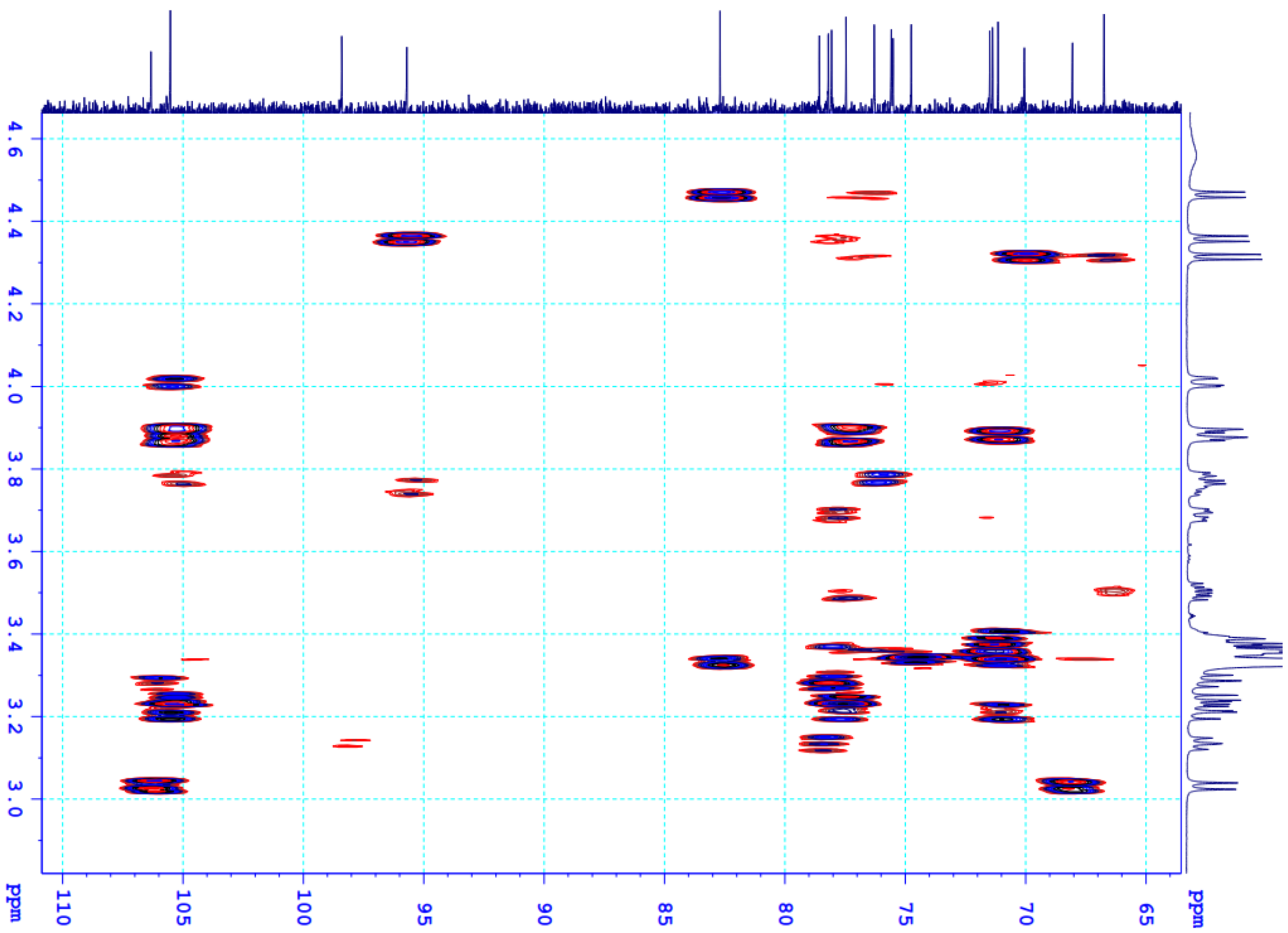

Figure S15. HMBC spectra of compound **2**

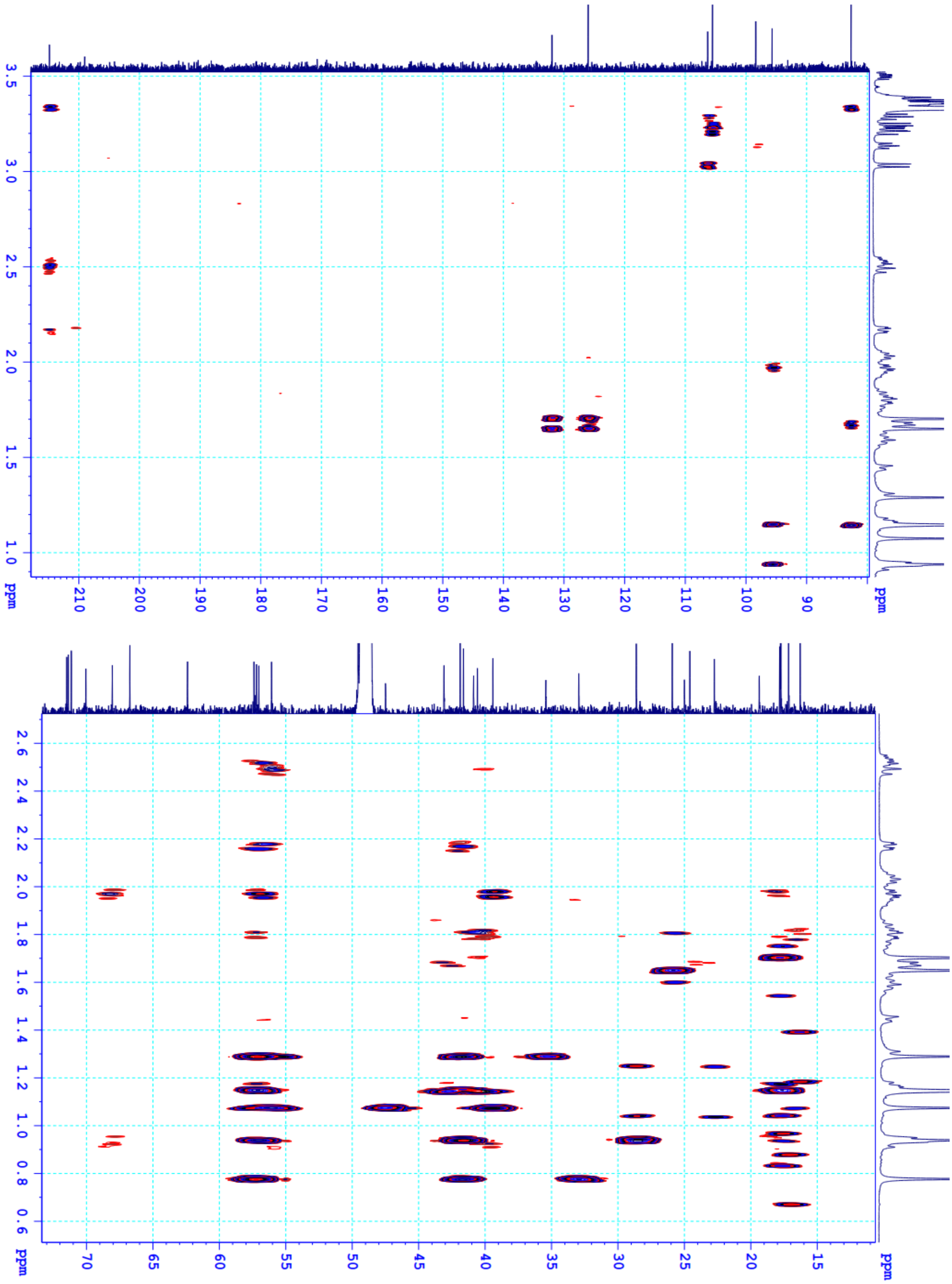

Figure S16. HMBC spectra of compound **2**

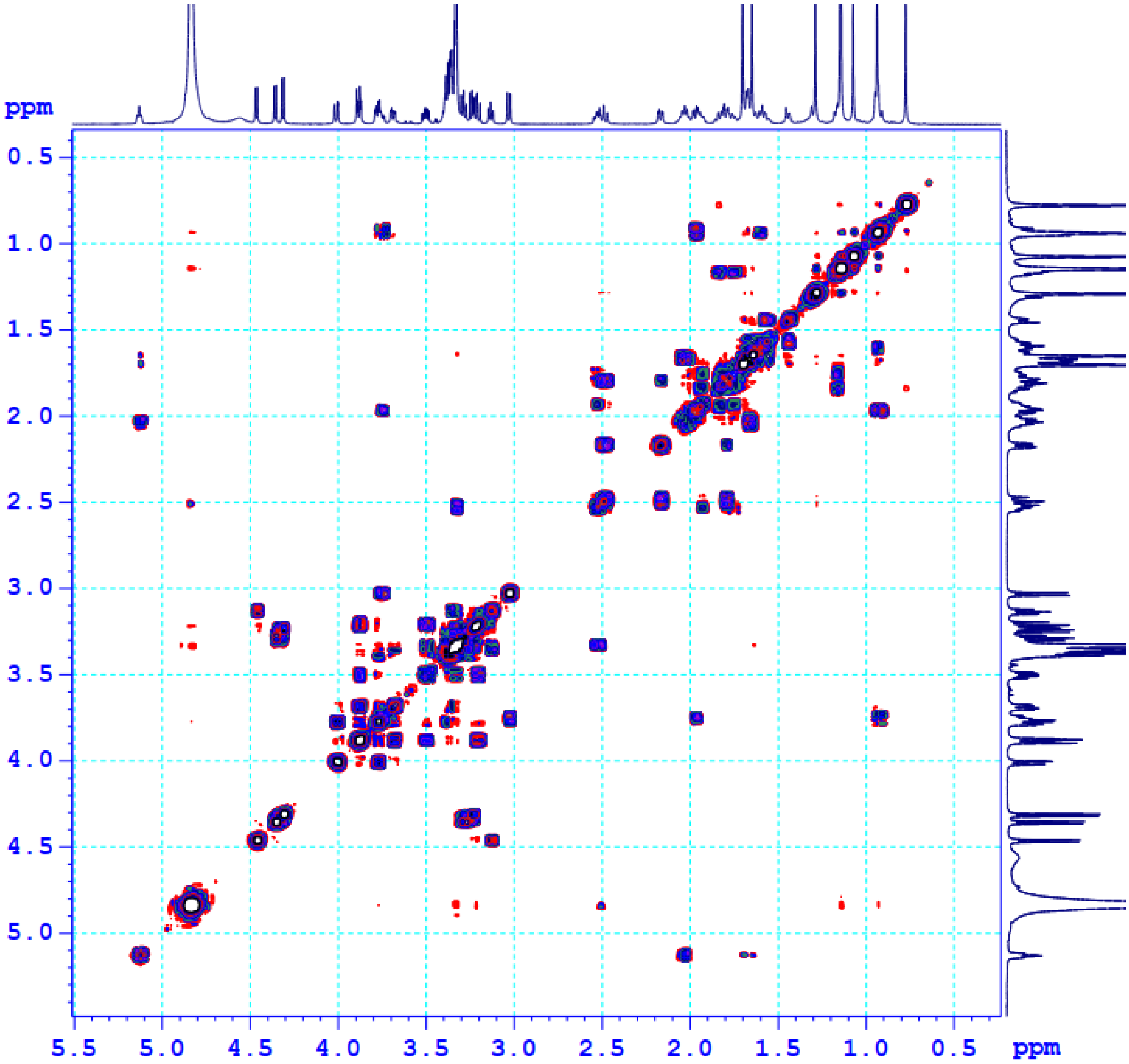

Figure S17. COSY spectrum of compound **2**

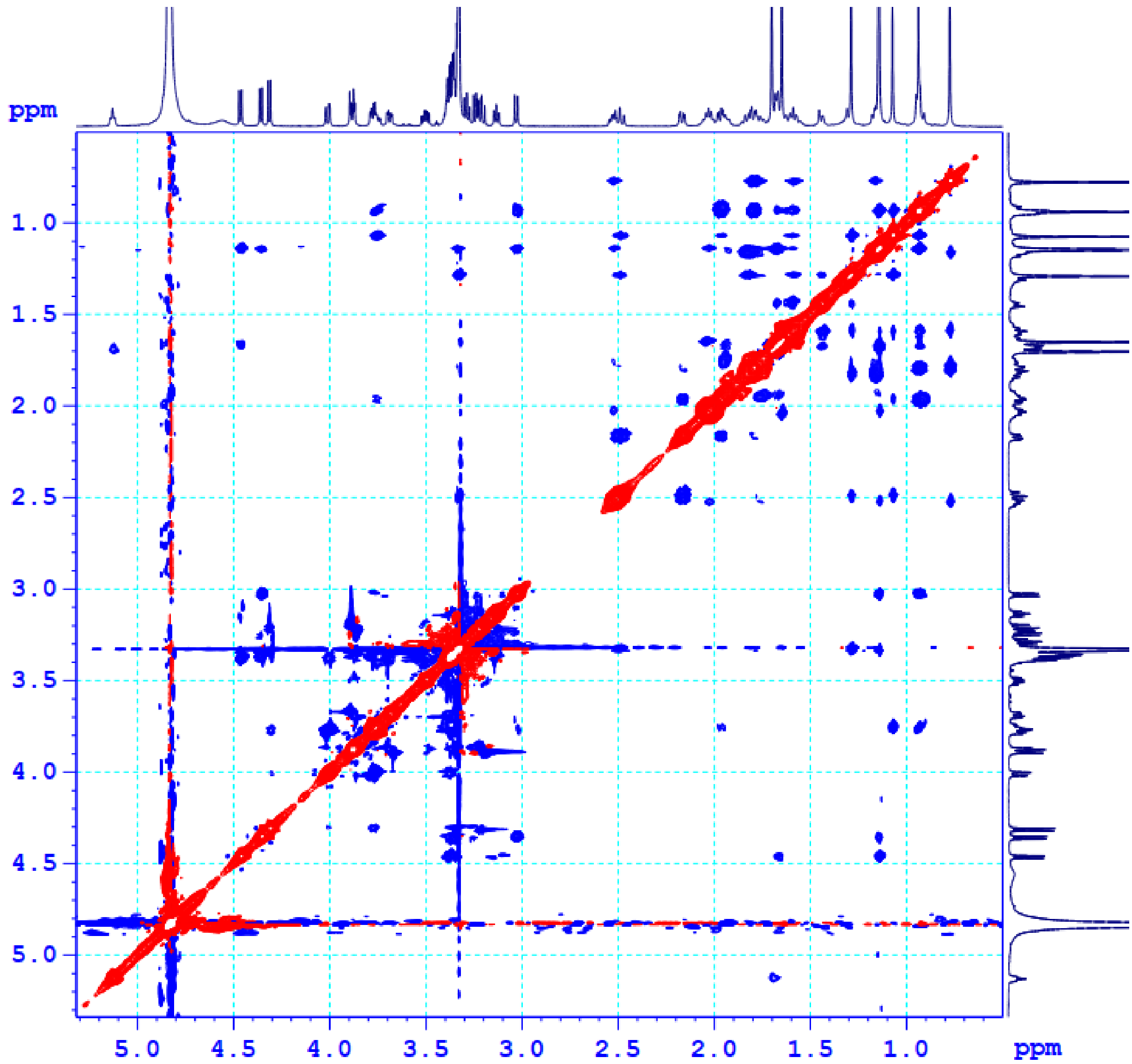

Figure S18. NOESY spectrum of compound **2**

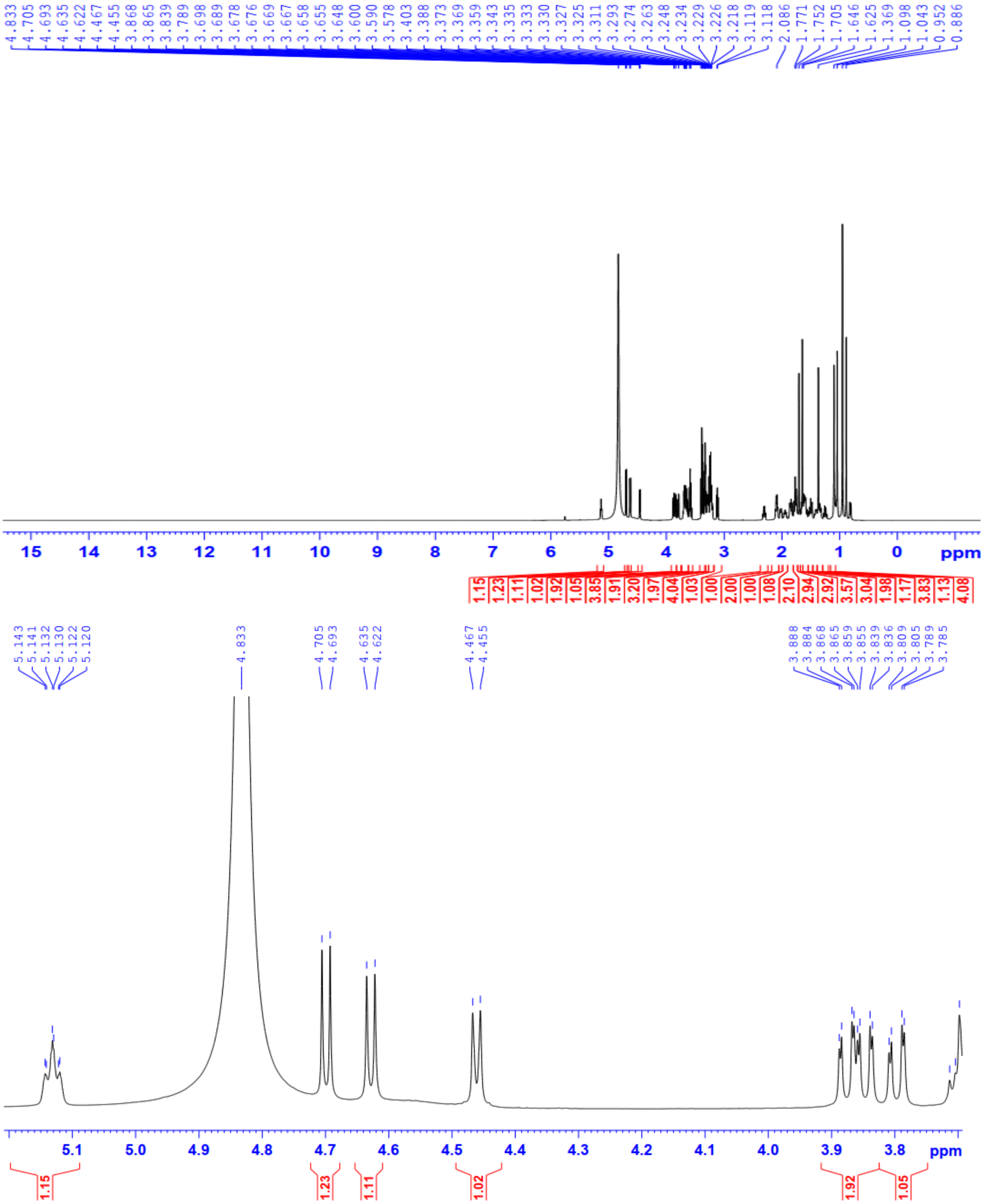

Figure S19. ^{1}H NMR spectra of compound **3**

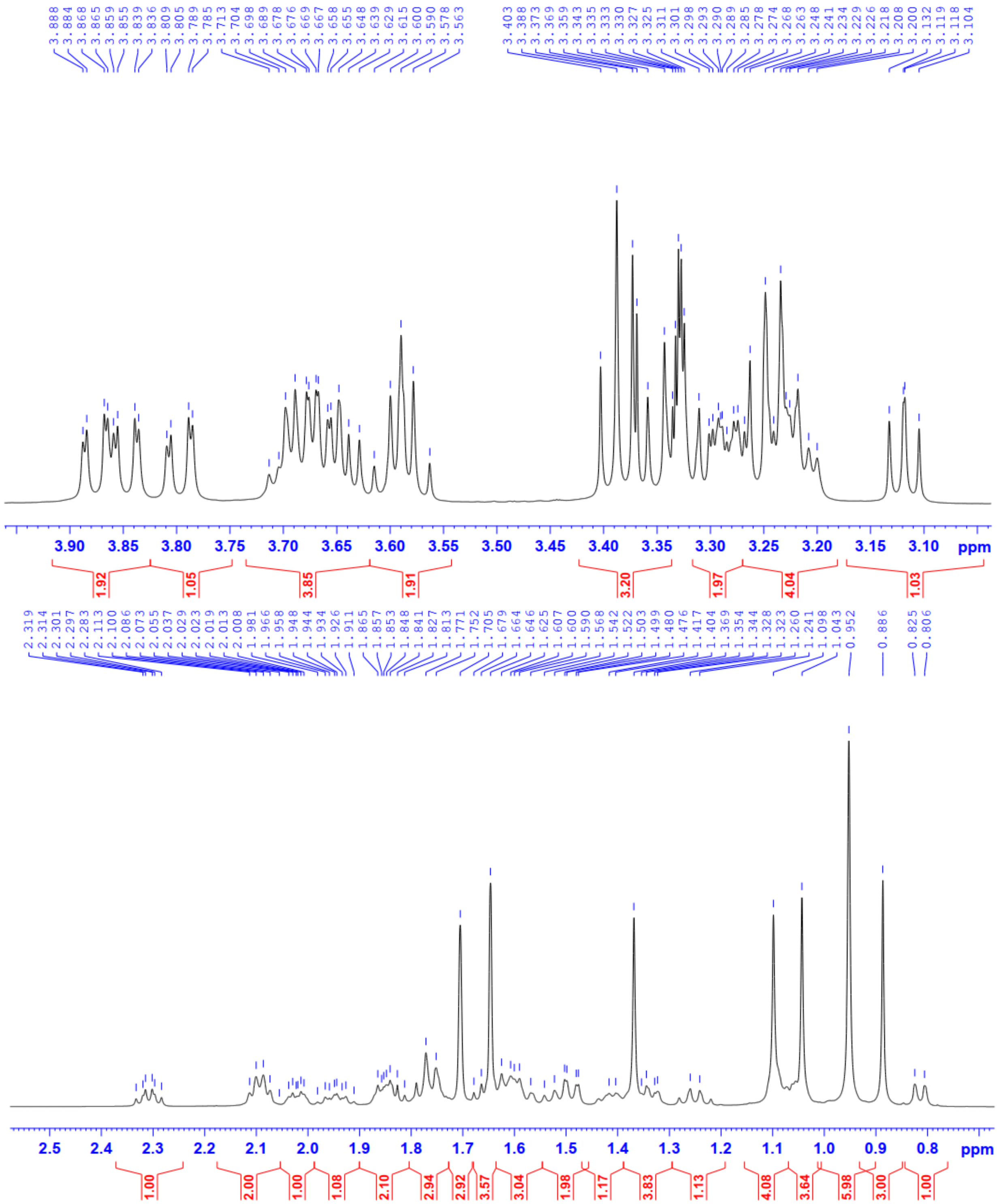

Figure S20. Expanded ^{1}H NMR spectra of compound **3**

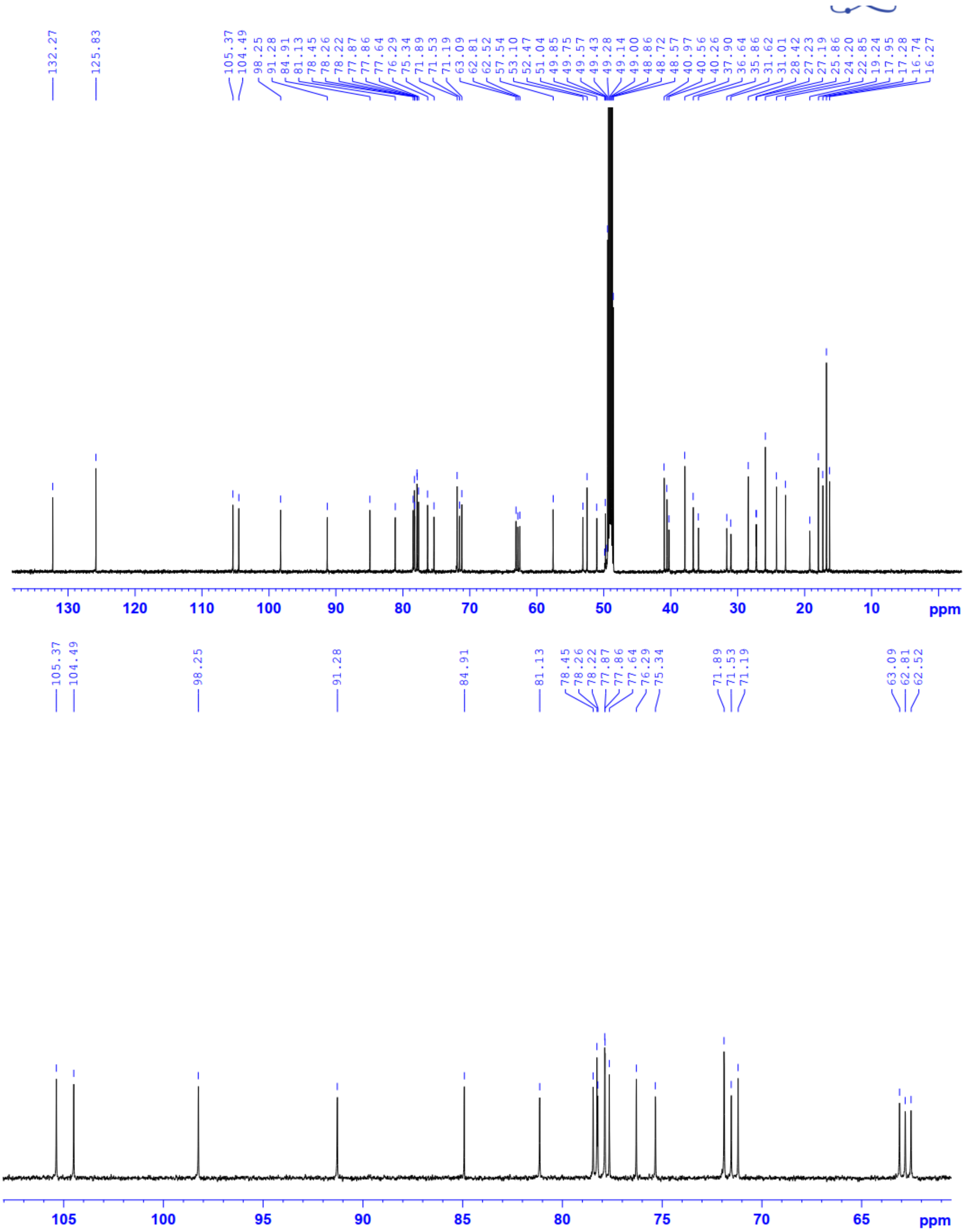

Figure S21. ^{13}C NMR spectra of compound **3**

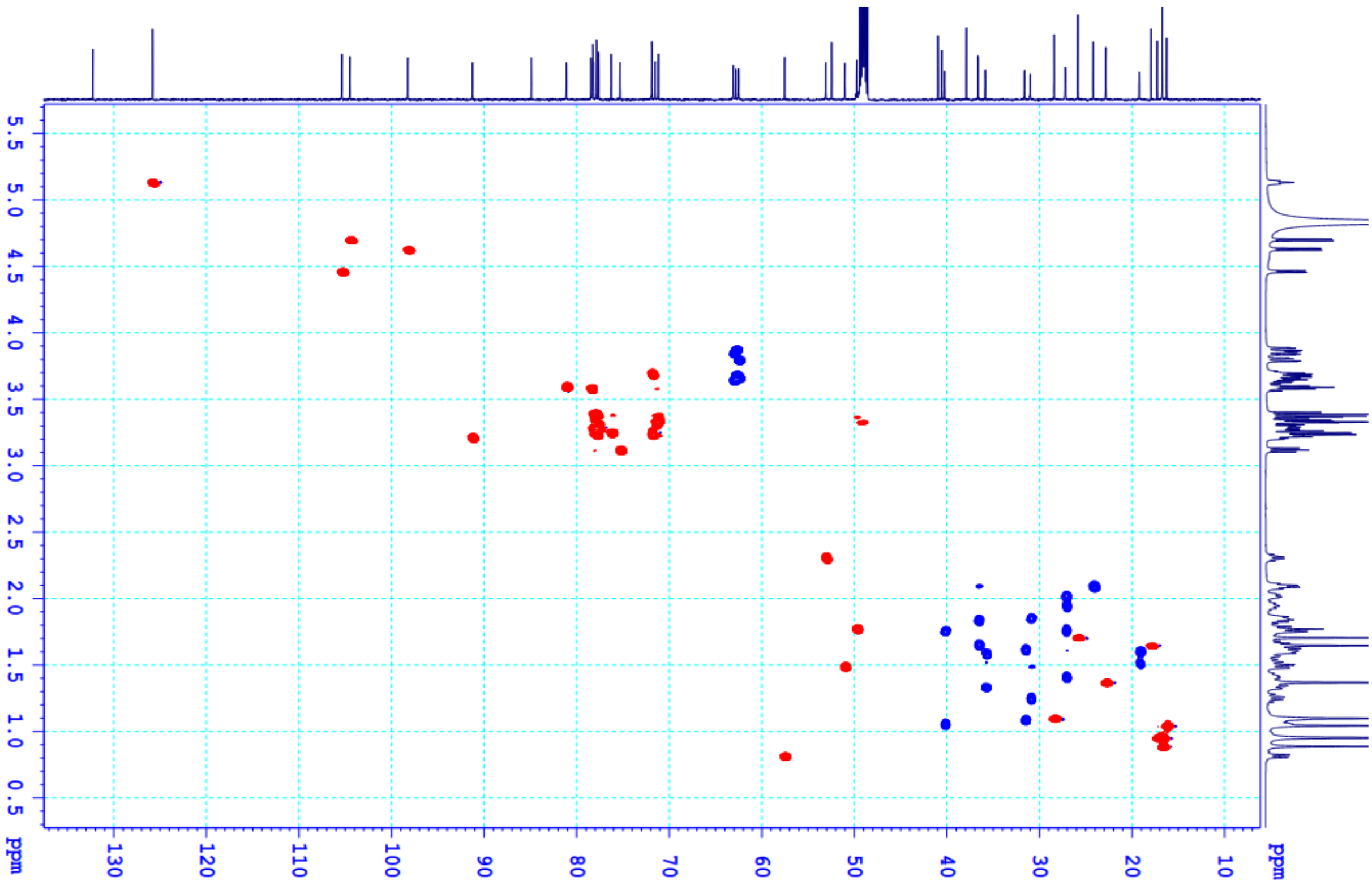

Figure S22. HSQC spectrum of compound **3**

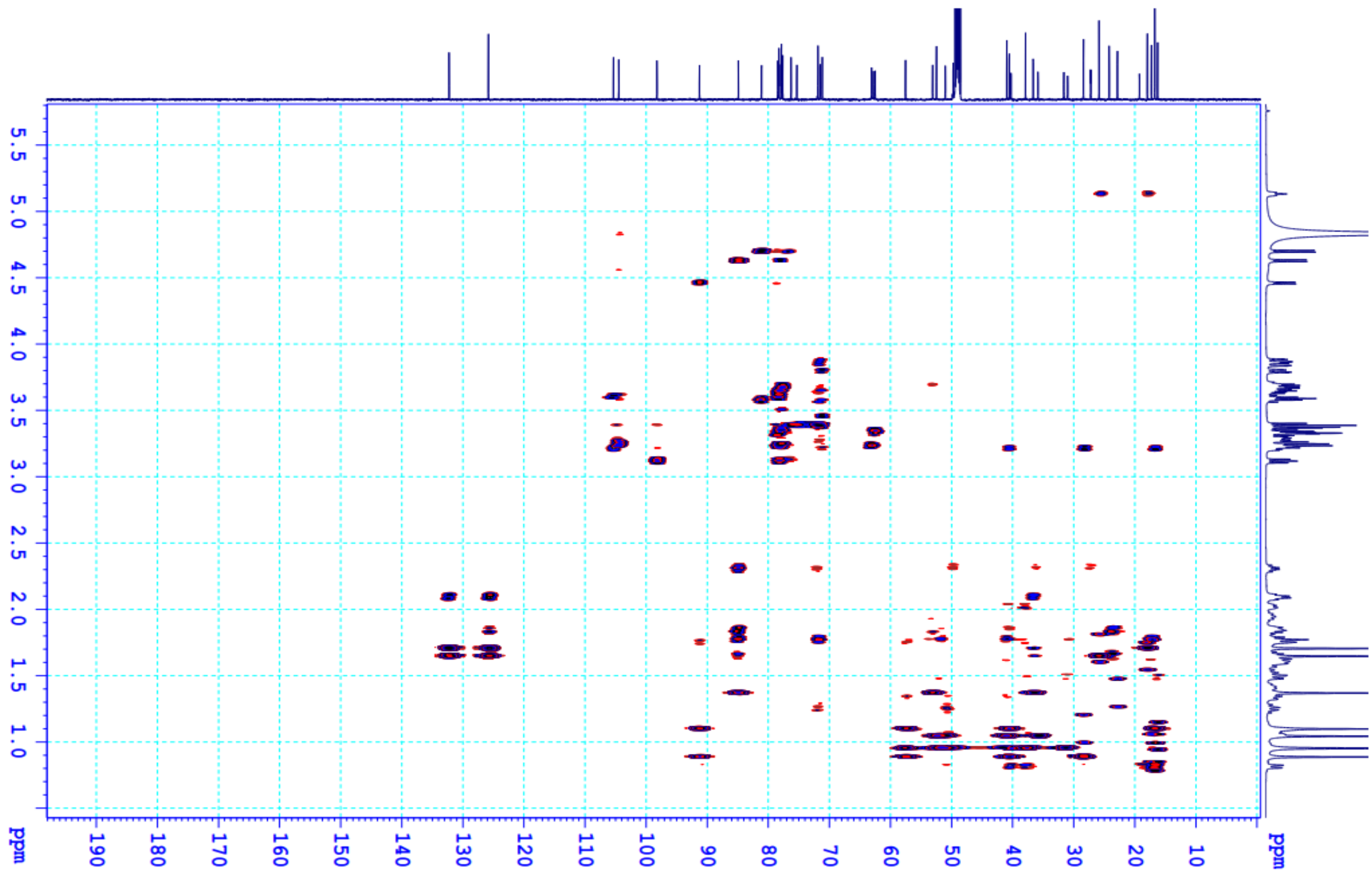

Figure S23. HMBC spectrum of compound **3**

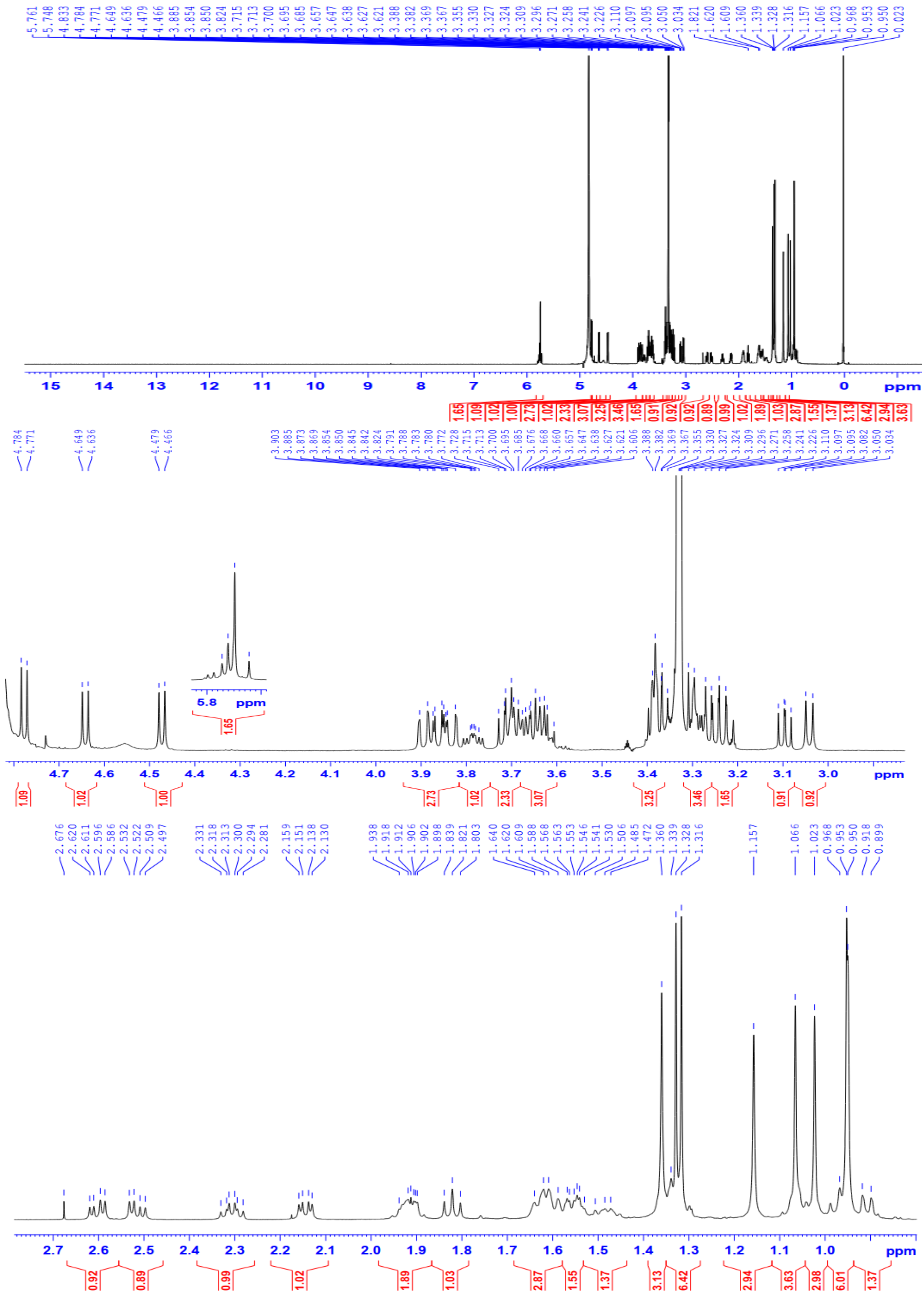

Figure S24. ^{1}H NMR spectra of compound **4**

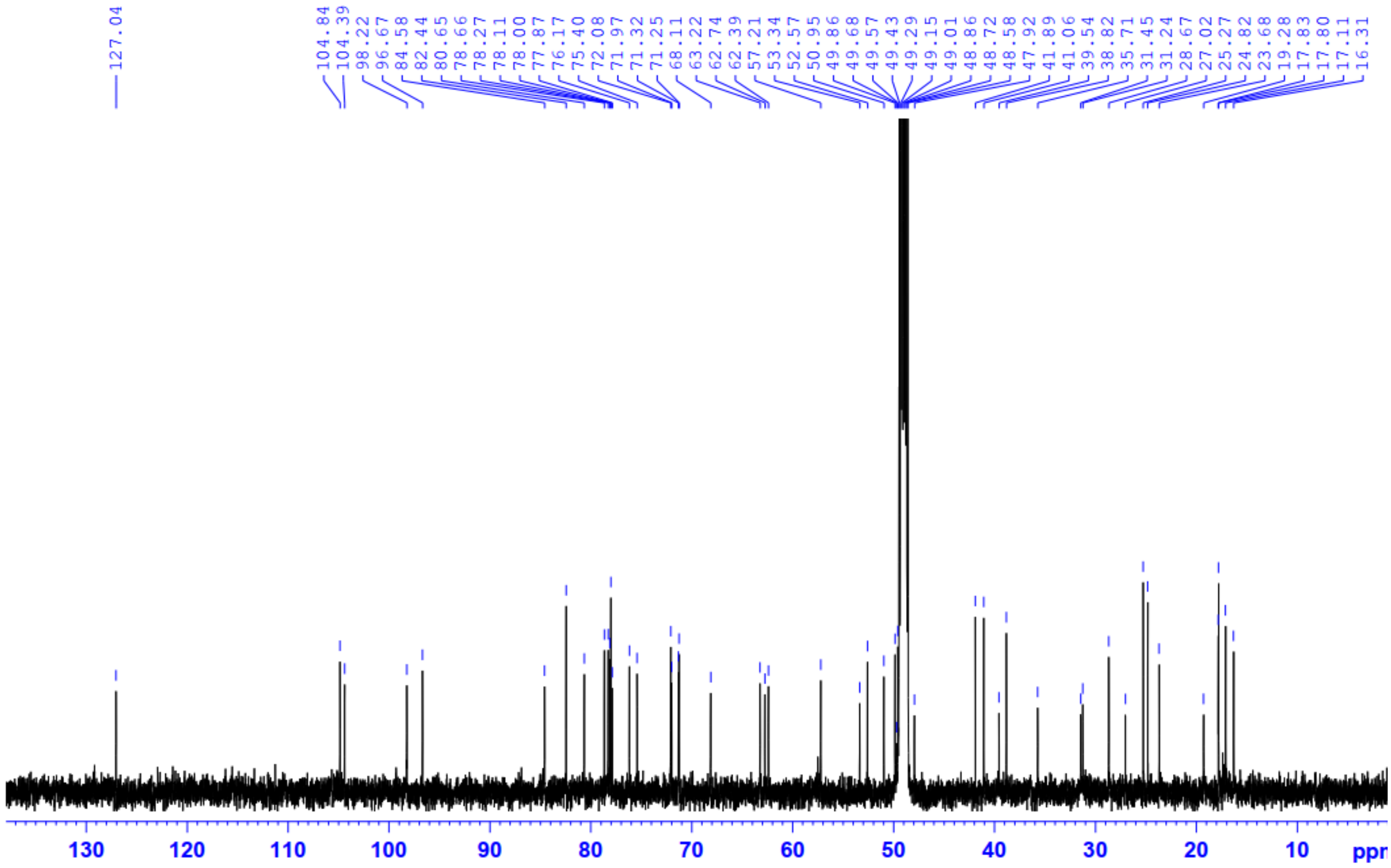

Figure S25. ^{13}C NMR spectrum of compound **4**

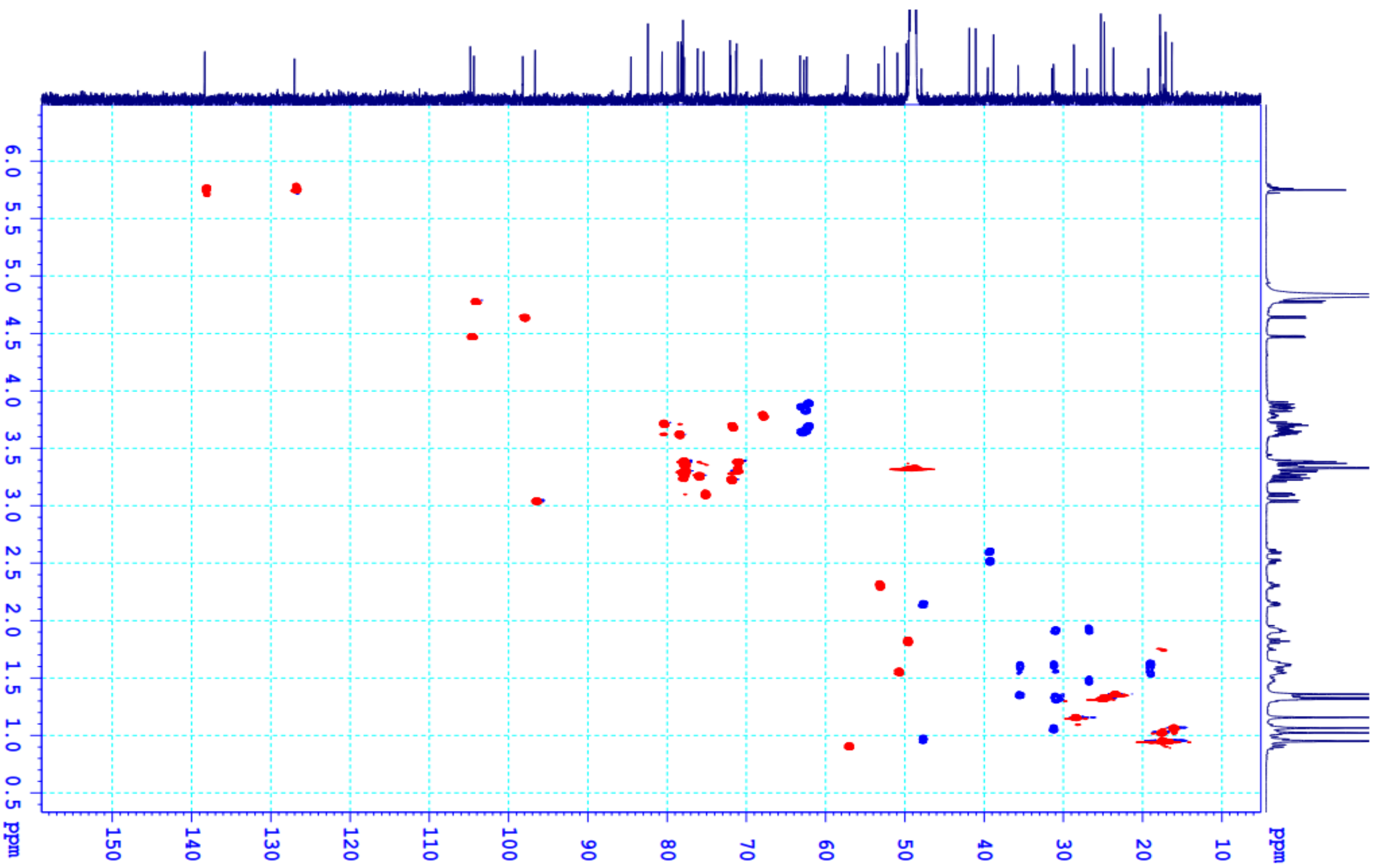

Figure S26. HSQC pectrum of compound **4**

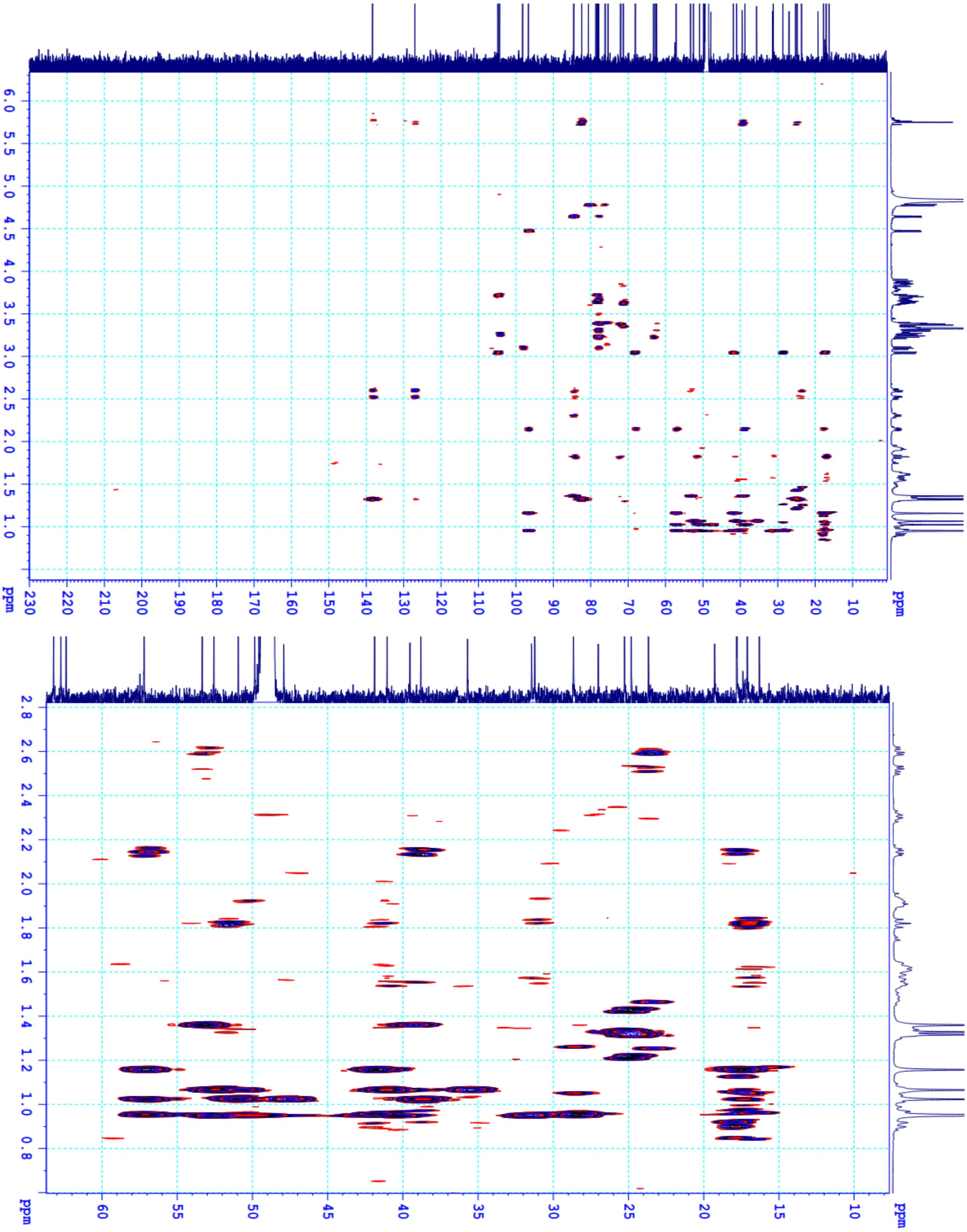

Figure S27. HMBC pectrum of compound **4**

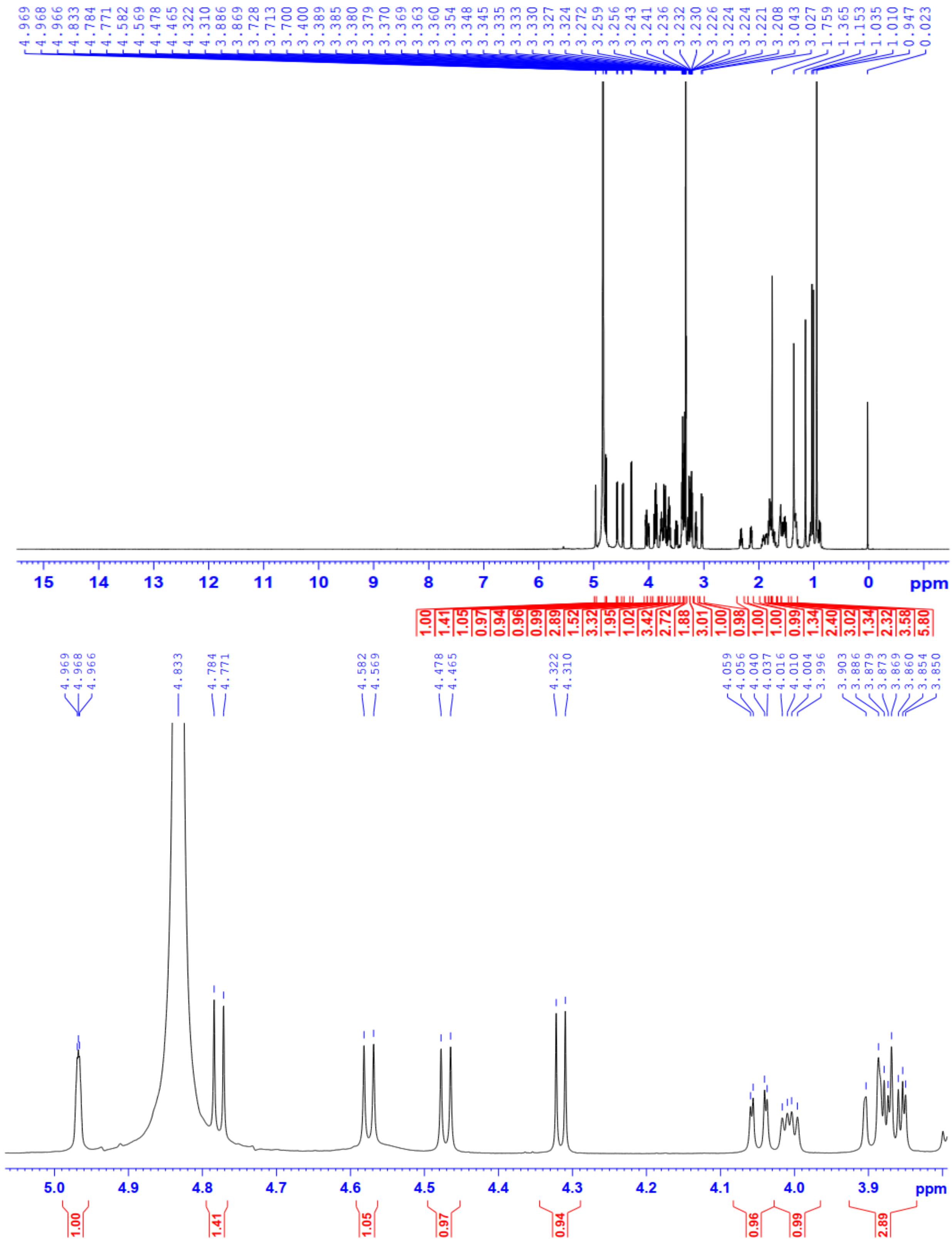

Figure S28. ^{1}H NMR spectra of compound **5**

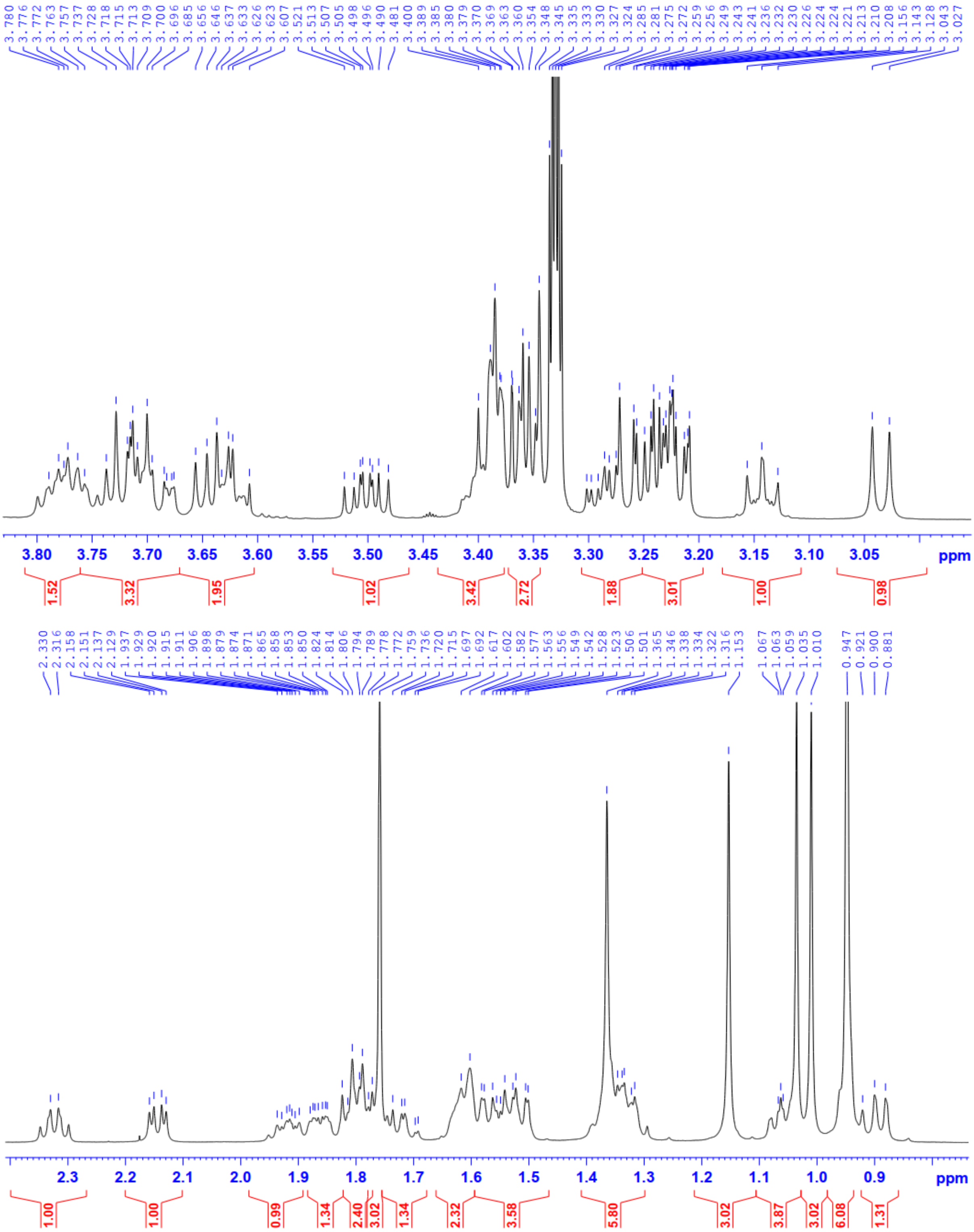

Figure S29. Expanded ^{1}H NMR spectra of compound **5**

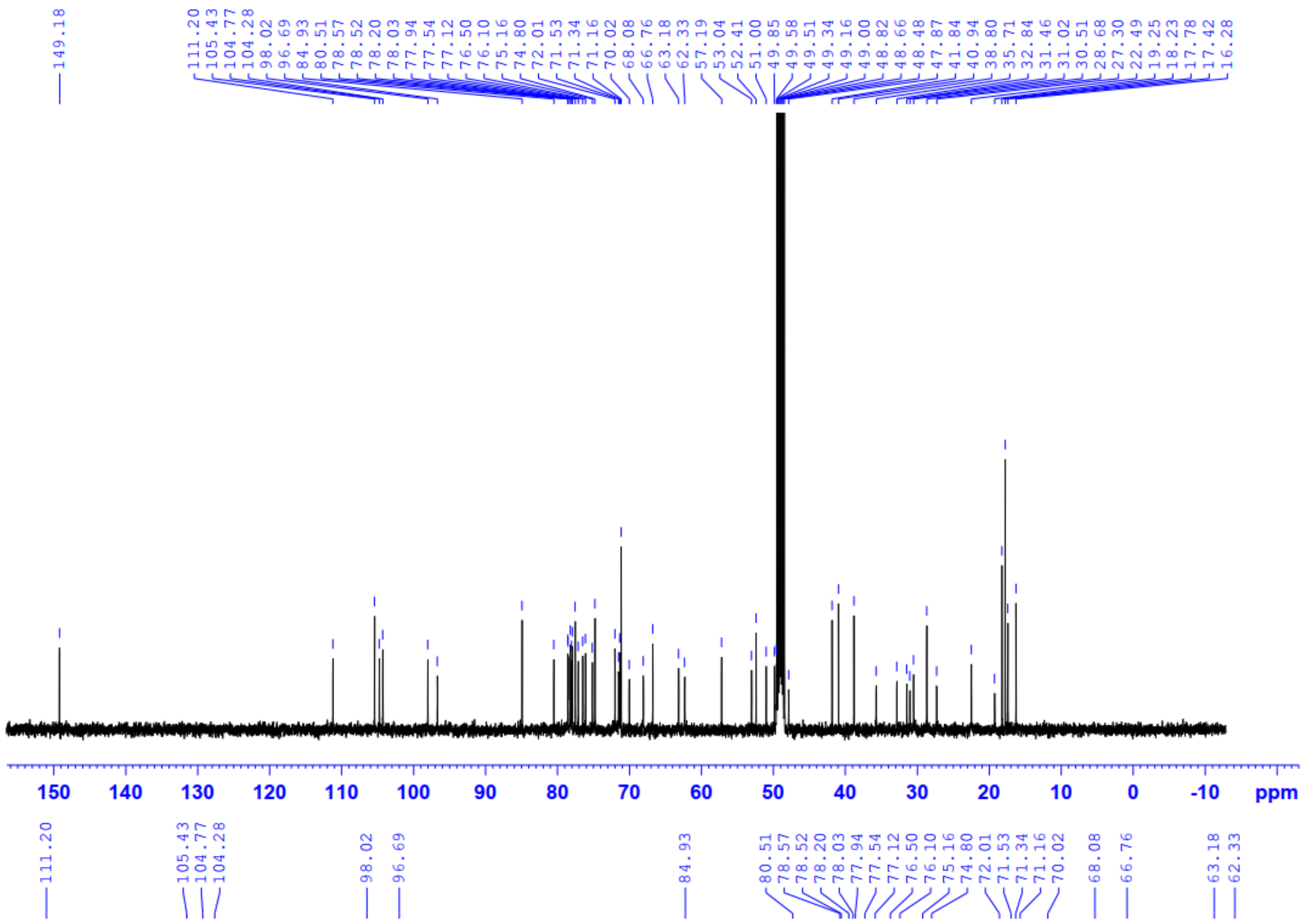

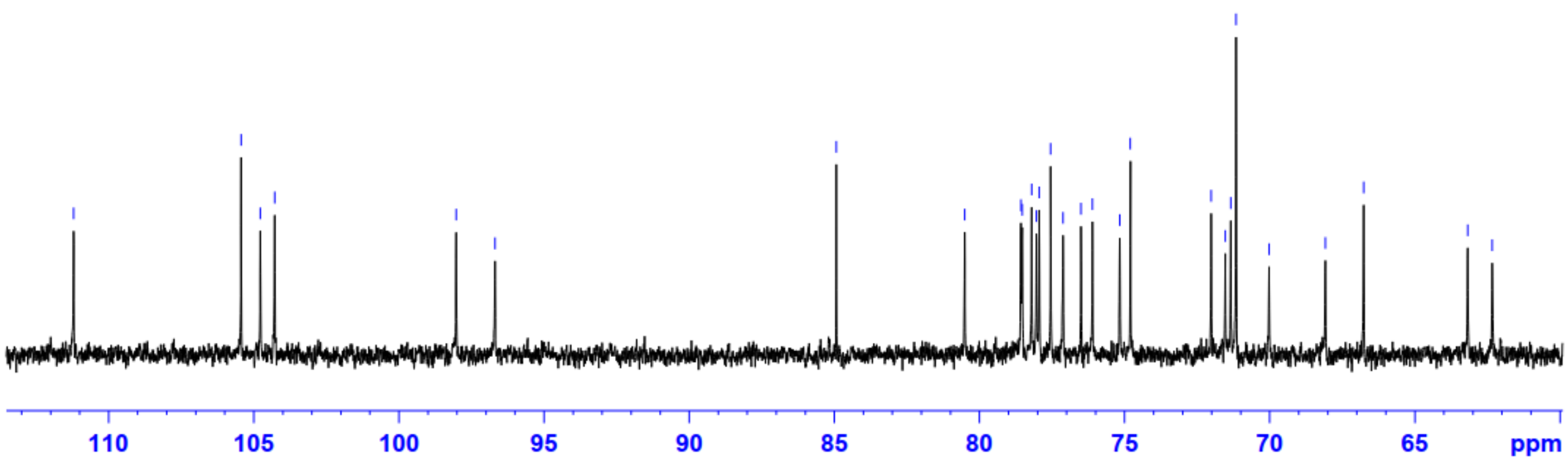

Figure S30. ^{13}C NMR spectra of compound **5**

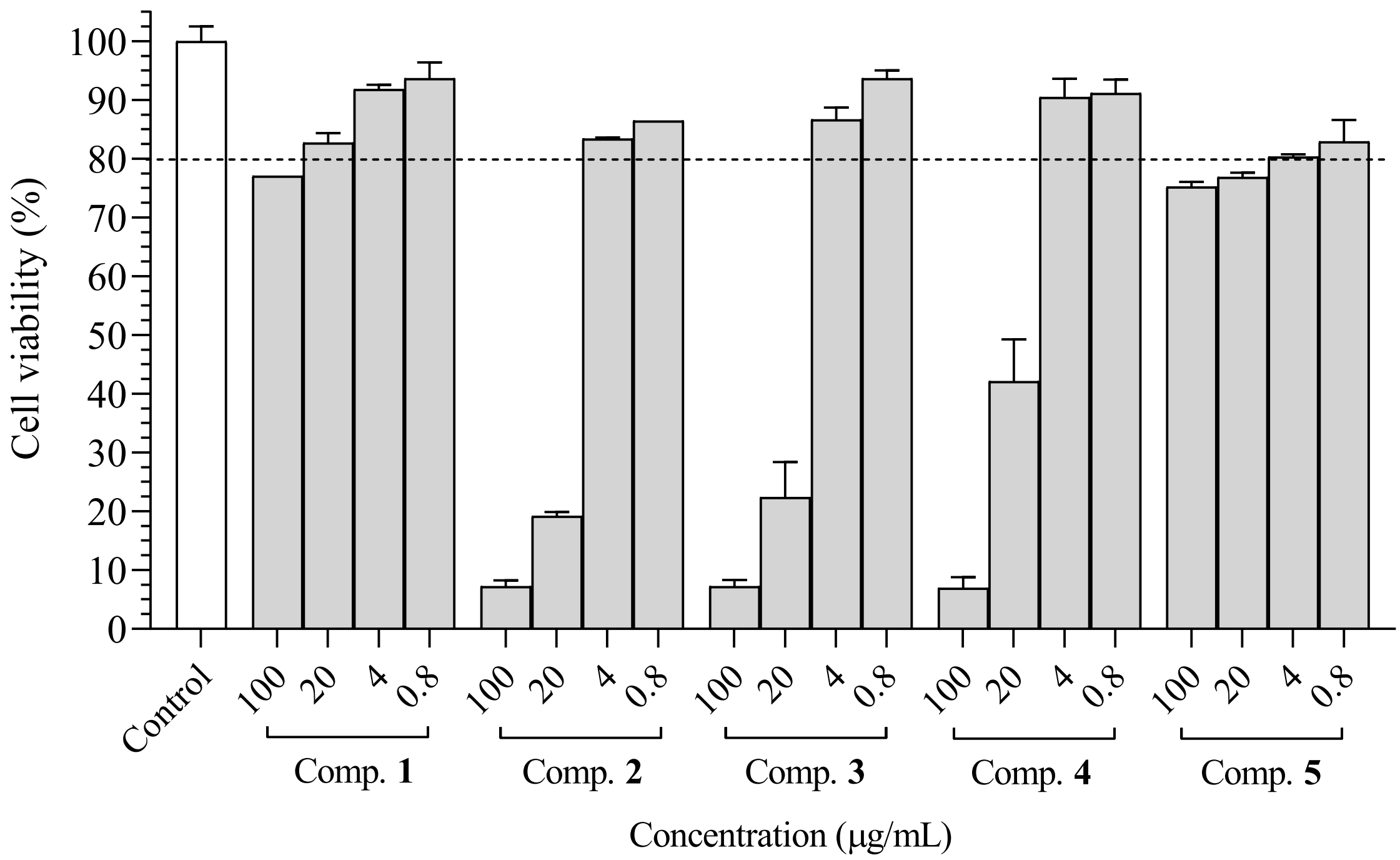

Figure S31. Effects of compounds **1-5** on the viability of MC3T3-E1 cells.

Table S1 NMR data of compound **3** in CD_3OD

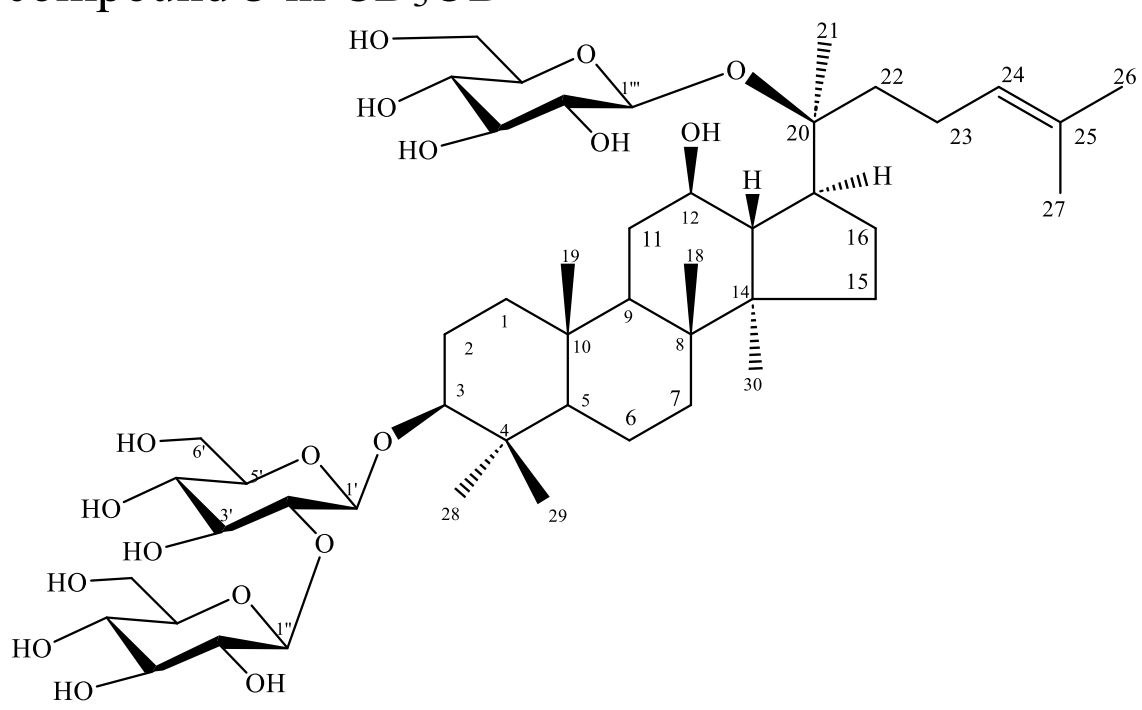

Ginsenoside Rd

C	δ_C[a,b]	δ_H[a,c] (mult., J = Hz)	C	δ_C[a,b]	δ_H[a,c] (mult., J = Hz)
1	40.3	1.03 (m)/1.75 (m)	3-O-glc		
2	27.2	1.75 (m)/2.01 (m)	1′	105.4	4.46 (d, 7.8)
3	91.3	3.21 (dd, 13.0, 4.8)	2′	81.1	3.59 (dd, 9.0, 7.8)
4	40.6	-	3′	78.5	3.58 (dd, 9.0, 9.0)
5	57.5	0.82 (br d, 11.4)	4′	71.9	3.23 (dd, 9.0, 9.0)
6	19.2	1.50 (m)/1.60 (m)	5′	77.9	3.37 (m)
7	35.9	1.33 (m)/1.59 (m)	6′	63.1	3.64 (dd, 12.0, 5.4)<br>3.84 (dd, 12.0, 1.8)
8	41.0	-	2′-O-glc		
9	51.0	1.48 (dd, 13.2, 3.0)	1″	104.5	4.70 (d, 7.8)
10	37.9	-	2″	76.3	3.25 (dd, 9.0, 7.8)
11	31.0	1.24 (m)/1.85 (m)	3″	78.2	3.27 (dd, 9.0, 9.0)
12	71.9	3.68 (m)	4″	71.5	3.35 (dd, 9.0, 9.0)
13	49.9	1.77 (dd, 10.8, 10.8)	5″	78.5	3.30 (m)
14	52.4	-	6″	62.8	3.68 (dd, 12.0, 5.4)<br>3.87 (dd, 12.0, 1.8)
15	31.6	1.08 (m)/1.61 (m)	20-O-glc		
16	27.2	1.40 (m)/1.95 (m)	1‴	98.3	4.63 (d, 7.8)
17	53.0	2.31 (m)	2‴	75.3	3.12 (dd, 9.0, 7.8)
18	16.3	1.04 (s)	3‴	78.3	3.30 (t, 9.0))
19	16.7	0.95 (s)	4‴	71.2	3.34 (t, 9.0)
20	84.9	-	5‴	77.6	3.29 (m)
21	22.9	1.37 (s)	6‴	62.5	3.67 (dd, 12.0, 5.4)<br>3.79 (dd, 12.0, 1.8)
22	36.6	1.64 (m)/1.83 (m)			
23	24.2	2.10 (q, 6.6)			
24	125.8	5.13 (t, 6.6)			
25	132.3	-			
26	25.9	1.71 (s)			
27	18.0	1.65 (s)			
28	28.4	1.10 (s)			
29	16.7	0.89 (s)			
30	17.3	0.95 (s)			

[a] CD_3OD, [b] 150 MHz, [c] 600 MHz

Table S2 NMR data of compound **4** in CD_3OD

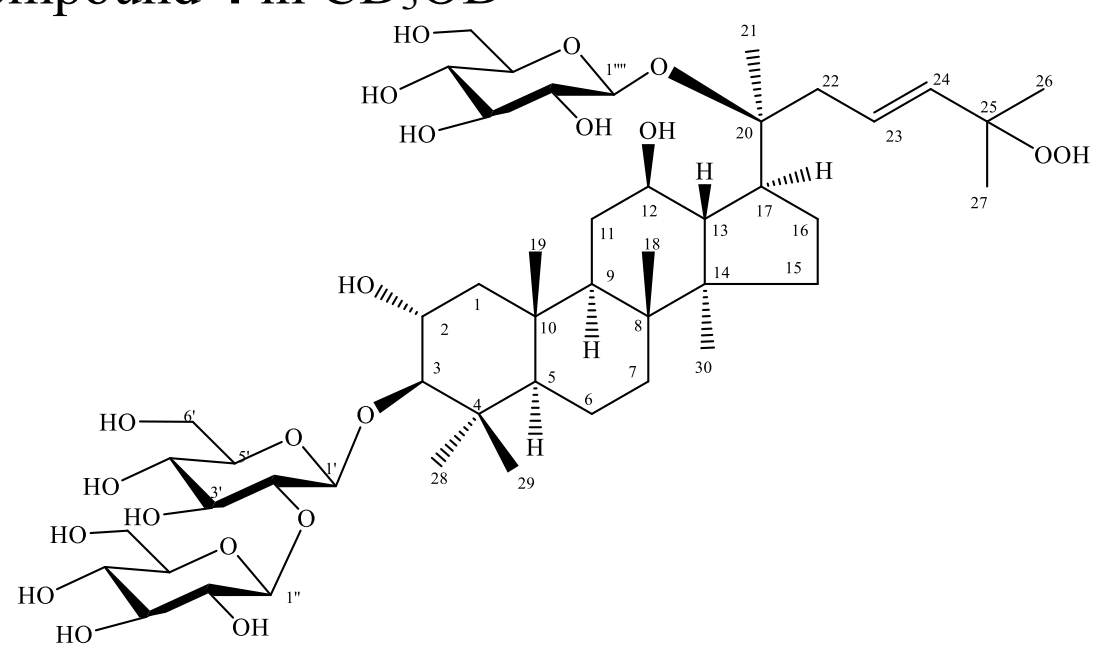

Gypenoside GD5

C	δ_C[a,b]	δ_H[a,c] (mult., *J* = Hz)	C	δ_C[a,b]	δ_H[a,c] (mult., *J* = Hz)
1	47.9	0.94-0.96 (m)<br>2.14 (dd, 12.6, 4.2)	3-O-glc		
2	68.1	3.78 (td, 9.0, 6.0)	1′	104.8	4.47 (d, 7.8)
3	96.7	3.04 (d, 9.0)	2′	80.7	3.72 (dd, 9.0, 7.8)
4	41.9	-	3′	78.7	3.62 (t, 9.0)
5	57.2	0.90 (br d, 12.0)	4′	71.3	3.38 (t, 9.0)
6	19.3	1.52-1.54 (m)<br>1.60-1.64 (m)	5′	77.9	3.27-3.30 (m)
7	35.7	1.34-1.36 (m)<br>1.58-1.62 (m)	6′	62.7	3.83 (dd, 12.0, 1.8)<br>3.65 (dd, 12.0, 5.4)
8	41.1	-	2′-O-glc		
9	51.0	1.52-1.56 (m)	1″	104.4	4.78 (d, 7.8)
10	38.8	-	2″	76.2	3.25 (dd, 9.0, 7.8)
11	31.5	1.05-1.07 (m)<br>1.59-1.61 (m)	3″	78.3	3.23 (t, 9.0)
12	72.0	3.66-3.70 (m)	4″	71.3	3.31 (t, 9.0)
13	49.6	1.80-1.84 (m)	5″	78.0	3.28-3.30 (m)
14	52.6	-	6″	63.2	3.64 (dd, 11.4, 5.4)<br>3.86 (dd, 11.4, 1.8)
15	31.2	1.33-1.35 (m)<br>1.88-1.91 (m)	20-O-glc		
16	27.0	1.47-1.49 (m)<br>1.89-1.93 (m)	1‴	98.2	4.64 (d, 7.8)
17	53.3	2.29-2.31 (m)	2‴	75.4	3.10 (dd, 9.0, 7.8)
18	16.3	1.07(s)	3‴	78.0	3.35 (t, 9.0)
19	17.8	1.02 (s)	4‴	72.1	3.22 (t, 9.0)
20	84.6	-	5‴	78.1	3.34-3.36 (m)
21	23.7	1.36 (s)	6‴	62.4	3.68 (dd, 12.0, 6.0)<br>3.88 (dd, 12.0, 2.4)
22	39.5	2.65 (dd, 13.8, 6.0)<br>2.51 (dd, 13.8, 7.2)			
23	127.0	5.77 (ddd, 15.6, 7.2, 6.0)			
24	138.4	5.73 (d, 15.6)			
25	82.4	-			
26	25.3	1.32 (s)			
27	24.8	1.33 (s)			
28	28.7	1.16 (s)			
29	17.8	0.95 (s)			
30	17.1	0.95 (s)			

[a] CD_3OD, [b]150 MHz, [c]600 MHz

Table S3 NMR data of compound **5** in CD_3OD

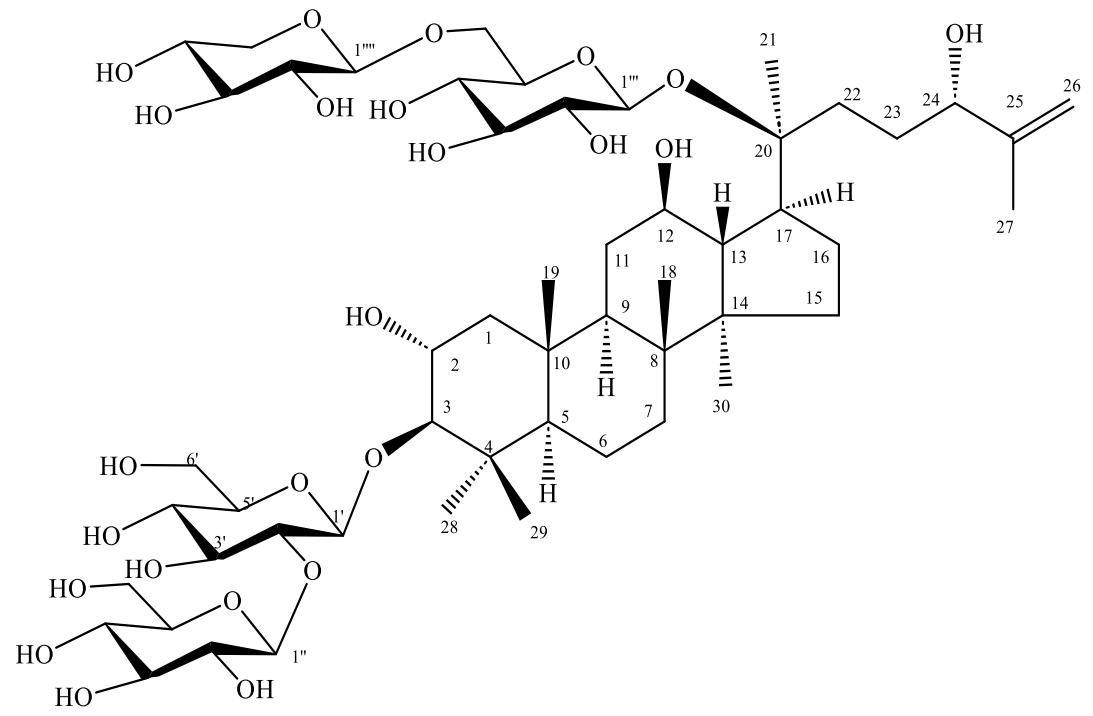

C	δ_C[a,b]	δ_H[a,c] (mult., *J* = Hz)	C	δ_C[a,b]	δ_H[a,c] (mult., *J* = Hz)
1	47.9	0.94-0.96 (m)/2.15 (dd, 12.6, 4.8)	3-O-glc		
2	68.1	3.76-3.79 (m)	1′	104.8	4.47 (d, 7.8)
3	96.7	3.04 (d, 9.6)	2′	80.5	3.71 (dd, 9.0, 7.8)
4	41.8	-	3′	78.5	3.62 (t, 9.0)
5	57.2	0.89 (br d, 12.0)	4′	71.3	3.23 (t, 9.0)
6	19.3	1.50-1.54 (m)/1.60-1.61 (m)	5′	78.0	3.20-3.22 (m)
7	35.7	1.42-1.46 (m)/1.56-1.60 (m)	6′	62.3	3.90 (dd, 12.0, 1.8) 3.70 (dd, 12.0, 5.4)
8	40.9	-	2′-O-glc		
9	51.0	1.50-1.53 (m)	1″	104.3	4.78 (d, 7.8)
10	38.8	-	2″	76.1	3.25 (dd, 9.0, 7.8)
11	31.2	1.34-1.36 (m)/1.90-1.92 (m)	3″	78.2	3.32 (t, 9.0)
12	71.8	3.70-3.74(m)	4″	71.2	3.28 (t, 9.0)
13	49.6	1.78-1.82 (m)	5″	77.5	3.28-3.30 (m)
14	52.5	-	6″	63.2	3.63 (dd, 11.4, 5.4) 3.87 (dd, 11.4, 1.8)
15	31.5	1.03-1.07 (m)/1.52-1.54 (m)	20-O-glc		
16	27.3	1.39-1.42 (m)/1.89-1.93 (m)	1‴	98.0	4.58 (d, 7.8)
17	53.0	2.31 (ddd, 9.0, 7.8, 7.2)	2‴	75.2	3.14 (dd, 9.0, 7.8)
18	16.3	1.03 (s)	3‴	78.6	3.35 (t, 9.0)
19	17.8	1.01 (s)	4‴	72.0	3.22 (t, 9.0)
20	84.9	-	5‴	76.5	3.37-3.42 (m)
21	22.5	1.36 (s)	6‴		
22	32.8	1.55-1.58 (m)/1.80-1.82 (m)	6‴-O-Xylose		
23	30.5	1.60-1.70 (m)	1⁗	105.4	4.32 (d, 7.2)
24	77.1	4.01 (dd, 7.2, 4.2)	2⁗	74.8	3.22 (dd, 9.0, 7.2)
25	149.2	-	3⁗	77.9	3.35 (t, 9.0)
26	111.2	4.97 (s)/4.82 (s)	4⁗	71.3	3.48-3.52 (m)
27	18.2	1.76 (s)	5⁗	66.8	3.87 (dd, 12.0, 5.0) 3.20 (dd, 12.0, 6.0)
28	28.7	1.15 (s)			
29	17.4	0.95 (s)			
30	17.8	0.95 (s)			

[a] *CD_3OD,* [b] *150 MHz,* [c] *600 MHz*
